# People devalue generative AI’s competence but not its advice in addressing societal and personal challenges

**DOI:** 10.1038/s44271-023-00032-x

**Published:** 2023-11-15

**Authors:** Robert Böhm, Moritz Jörling, Leonhard Reiter, Christoph Fuchs

**Affiliations:** 1 Faculty of Psychology, University of Vienna, Universitätsstrasse 7, 1010 Vienna, Austria; 2 Department of Psychology and Copenhagen Center for Social Data Science (SODAS), University of Copenhagen, Øster Farimagsgade 2A, 1353 Copenhagen K, Denmark; 3 Marketing Department, EMLyon Business School, 23 Av. Guy de Collongue, 69130 Écully, France; 4 Faculty of Business, Economics, and Statistics, University of Vienna, Oskar-Morgenstern-Platz 1, 1090 Vienna, Austria

**Keywords:** Human behaviour, Information systems and information technology

## Abstract

The release of ChatGPT and related tools have made generative artificial intelligence (AI) easily accessible for the broader public. We conducted four preregistered experimental studies (total *N* = 3308; participants from the US) to investigate people’s perceptions of generative AI and the advice it generates on how to address societal and personal challenges. The results indicate that when individuals are (vs. are not) aware that the advice was generated by AI, they devalue the author’s competence but not the content or the intention to share and follow the advice on how to address societal challenges (Study 1) and personal challenges (Studies 2a and 2b). Study 3 further shows that individuals’ preference to receive advice from AI (vs. human experts) increases when they gained positive experience with generative AI advice in the past. The results are discussed regarding the nature of AI aversion in the context of generative AI and beyond.

## Abstract (web summary)

When people receive advice written by large language models, they downrate the competence of the source when they know the source isn’t human. Their preference to receive advice by large language models increases with positive experience.

## Introduction

The growing integration of artificial intelligence (AI) into various aspects of our lives presents a remarkable opportunity to address both societal and personal challenges. AI’s potential lies not only in its ability to analyze data but also in its capacity to provide intelligent suggestions to tackle complex problems. In the realm of societal challenges, such as climate change and pandemic preparedness, AI can contribute by offering proactive recommendations for sustainable practices and efficient response strategies. Additionally, in the context of personal challenges like improving healthy eating and saving money, AI can act as a personalized advisor, providing tailored suggestions and guidance. By leveraging AI’s capability to generate intelligent suggestions, we can empower individuals and societies to navigate these challenges more effectively, fostering sustainable behavior and improving overall well-being.

This introductory paragraph was entirely written by ChatGPT (based on Generative Pre-Trained Transformer 3.5). ChatGPT is a publicly accessible, state-of-the-art language generation model developed by OpenAI (https://chat.openai.com/). Since its release in November 2022, ChatGPT has garnered considerable attention and enthusiasm for its capabilities and potential applications, both in research and everyday use. For instance, it has been stated that “the potential impact of this technology is mind-boggling” and “will transform our lives”^1^; it will “potentially contribute to some of the world’s more complex issues, such as education, health, and climate change”^2^.

However, to fully realize the potential of AI in addressing societal and personal challenges, its users and potential beneficiaries (humans) need to accept and adopt AI-generated recommendations in the first place^3^. Now ask yourself: Would you evaluate the introductory paragraph and its author differently when you know (vs. do not know) that it has been written by an AI (vs. a human expert)? This is the very question we answer in this research, motivated by the fact that AI-generated content—whether identifiable to the readers as written by AI or not—will enter more and more areas of daily life. Specifically, we aim to contribute to a deeper understanding of the potential use and barriers to the adoption of publicly accessible AI, such as ChatGPT, in helping humanity solve pressing societal and personal challenges.

Although the available evidence is rather limited, prior research indicates that people are not particularly good at differentiating between AI- versus human-generated content (e.g., stories, news articles, recipes, poems) when the author identity is not transparent—even before recent improvements in generative AI such as GPT 3.5 and GPT 4 were built into ChatGPT^4,5^. Only recently have social and behavioral scientists started to evaluate the quality of responses by the new generation of generative AI, and people’s perception thereof. In this vein, research has demonstrated that people are not able to detect AI-generated self-presentations across different contexts like dating, hospitality, and professional interactions^6^. More specifically, an analysis of language features revealed that humans overly rely on intuitive but misleading heuristics like the usage of first-person pronouns or the discussion of family topics in a human manner, making human evaluation of AI-generated language predictable and manipulable. Other reports indicate that even experts cannot always distinguish between human- and AI-generated content. For example, when given a mixture of original and ChatGPT-generated medical scientific abstracts, blinded medical researchers could identify only 68% of the ChatGPT-generated abstracts as fabricated^7^. Other research evaluated the performance of ChatGPT on the United States Medical Licensing Exam (USMLE) and found that the tool performed near or at the passing threshold^8^. Taken together, there is anecdotal evidence that new generative AI such as ChatGPT generates content that is similar to and can hardly be discriminated from human-generated content.

But how do people *evaluate* content when they know that it has been generated by an AI? Much of the research in this domain has focused on studying individual preferences for AI/algorithm- versus human-generated advice for different tasks. An increasing body of research suggests that people tend to prefer human advice over AI advice and are less likely to follow the latter, an effect often referred to as ‘algorithm aversion’ (for comprehensive literature reviews, see^9–11^). Studies have demonstrated that individuals are (more) averse to following AI/algorithmic support in moral decision contexts^12,13^, in medical decision contexts^14,15^, and in hedonic contexts^16^. The findings of some studies, however, suggest that there is no general aversion against algorithmic decision support, but rather that its adoption is dependent on the specific application context^17^. For example, some research found that people follow dishonesty-promoting advice generated by an AI as much as they follow such advice that was given by a human^18^. To reconcile this inconsistency, it has recently been proposed that the aversion toward using AI/algorithmic advice depends on the identity relevance of the respective context^19^, with some studies even showing an appreciation of algorithmic advice in contexts with low identity relevance, such as numerical estimation or forecasting tasks^20^.

Adding to this literature, our main contribution is to provide a systematic investigation of people’s evaluations of advice by generative AI and their willingness to receive such advice. We focus on advice in contexts that have some identity relevance to the evaluators. That is, we investigate recommendations to societal challenges (Studies 1 and 3) and personal challenges (Studies 2a and 2b) when evaluators are (vs. are not) aware of the author identity. We assess several outcome criteria, including evaluations of the author and the content, as well as its potential downstream consequences, such as the choice between receiving AI- vs. human-generated advice.

## Methods

All studies involved human participants. The study procedures followed the national ethical regulations. Additionally, the studies received approval from the ethical review board of the Department of Occupational, Economic, and Social Psychology at the University of Vienna (protocol numbers #2022_W_004A and #2023_W_002A). All participants provided informed consent. Participants’ gender was self-selected. Participants received financial remuneration for their study participation, which was paid via the panel provider. The size of remuneration was chosen such that the hourly payment roughly matched 8 British Pounds.

### Study 1 design and experimental factors

Study 1 included three treatment variations in a 2 (author identity: AI vs. human author) × 2 (author transparency: transparent vs. non-transparent) × 5 (context: fake news vs. migration vs. global warming vs. pandemic preparedness vs. future workforce) between-participants design. Participants were randomly assigned to the conditions.

Firstly, we manipulated the author identity of the text providing the recommendation on how to solve societal challenges, which was either a ChatGPT or a human expert (see the “Generative AI content” section). Secondly, we manipulated whether participants knew the identity of the author. For the author transparency condition, in the case of a human author, we referred to a “human expert” across contexts; to match the expert framing in the case of an AI author, we referred to a “knowledgeable artificial intelligence bot.” In the non-transparent condition, we simply stated that “the following response has been proposed.” Lastly, to ensure generalizability across societal challenges, we compared recommendations for five grand societal challenges—fake news, global warming, pandemic preparedness, effect of AI on the workforce, and refugee reception.

### Study 1 participants

An a-priori power analysis using G*Power^21^ for an Analysis of Variance (ANOVA) with Bonferroni-adjusted *p*-values (avoiding alpha inflation due to the three dependent variables) resulted in a minimum sample size of 920 to detect small-to-medium effects of Cohen’s *f* = 0.2 with a large power of 0.95. We therefore preregistered to recruit 1000 participants. The final sample consists of *N* = 1003 participants from the US. There were 488 (50%) men, 496 (49%) women, and 19 (1%) participants choose another gender option. Their mean age was *M* = 41 (SD = 14) and 62% of the participants had at least a bachelor’s degree.

### Studies 2a and 2b design and experimental factors

Different from Study 1, we only included recommendations proposed by an AI as we were mainly interested in the devaluation of AI-generated responses when the author identity was transparent versus non-transparent. To this end, both Studies 2a and 2b used a 2 (author transparency: transparent vs. non-transparent) × 6 (context: start exercising more regularly vs. quitting smoking vs. eating healthier vs. reducing time spent on the phone vs. saving money vs. having a positive impact on the world) between-participants design. Participants were randomly assigned to the author transparency condition. Context was quasi-experimentally self-selected based on the participant’s personal preference in Study 2a and randomly assigned in Study 2b.

Author transparency was manipulated as in Study 1. The contexts included in this study were exercising more regularly, quitting smoking, eating healthier, reducing time spent on the phone, saving money, and having a positive impact on the world. In Study 2a, participants were presented with all challenges and could select a context that was most important for themselves. Our reasoning was that when people choose a context personally relevant to them, they are likely more motivated to accurately evaluate the stimulus materials. However, self-selection also creates the problem of endogeneity bias. Therefore, Study 2b was a replication study with the only difference that participants were randomly allocated to one of the contexts.

### Study 2a and 2b participants

In Study 2a, as a result of Study 1, we expected an effect of *f* = 0.17 for the transparency manipulation on the author evaluation. An a-priori power analysis for an ANOVA using G*Power^21^ with a power of 0.95 an alpha level of 0.05 recommended a sample size of at least 452. We preregistered to recruit at least 500 participants. Study 2a’s final sample consists of *N* = 501 US participants. Of those, 246 (49%) were men, 247 (49%) were women, and 8 (2%) chose another gender option. The mean age was *M* = 40 (SD = 15) and 65% of participants had at least a bachelor’s degree.

In Study 2b, we expected a somewhat smaller effect of *f* = 0.1 than in Study 2a for the transparency manipulation on the perceived author competence, as in Study 2b the context was assigned exogenously and participants might therefore be less attentive to the study materials. An a-priori power analysis for an ANOVA using G*Power^21^ with a power of 0.8 an alpha level of 0.05 recommended a sample size of at least 788; we preregistered to recruit 800 participants. Study 2b’s final sample consists of *N* = 800 US participants. Of those, 395 (49%) were men, 389 (49%) were women, and 16 (2%) chose another gender option. The mean age was *M* = 40 (SD = 14) and 60% of participants had at least a bachelor’s degree.

### Study 3 design and experimental factors

This study applied a 2 (transparency of author identity in prior experience: transparent vs. non-transparent) × 3 (context: refugee helping vs. global warming vs. pandemic preparedness) between-participants design. Participants were randomly assigned to the experimental conditions.

All participants were exposed to two recommendations for addressing a societal challenge by either a human expert or an AI in a randomized order. The context and corresponding content of advice by both authors was held constant across conditions, i.e., tackling fake news (see Study 1 and the “Stimuli” section in the Supplementary Information). Importantly, only after participants have read both recommendations and evaluated their quality, half of the participants were informed about which advice was from an AI author and which was from a human author. The other half of the participants received no information about the author identity.

We used three contexts of societal challenges to choose an advisor for how to address them, using the same materials as in Study 1, i.e., global warming, pandemic preparedness, and refugee reception (see Study 1 and the “Stimuli” section in the Supplementary Information). These contexts were chosen because in Study 1 there was a greater perceived competence of the human compared to the AI advisor when people knew the identity of the advisor (simple effect of author identity in these three contexts when the author identity was known as in Study 3: *F*(1, 265) = 8.12, *p* = 0.005, Cohen’s *f* = 0.18). Importantly, participants chose the advisor while knowing the context but without knowing the specific recommendation given by the advisor in this context.

### Study 3 participants

Following an a-priori power analysis using G*Power^21^ to test the first hypothesis (i.e., a higher rate of choosing an AI advisor when the author identity was transparent in prior experience, see below) with an ANOVA, a power of 0.80 to detect a small effect (OR = 1.5) with an alpha level of 0.05, resulted in a minimum sample size of 936 participants. We therefore aimed to recruit 1000 participants. The final sample consists of *N* = 1004 US participants. Of those, 496 (50%) were men, 487 (49%) were women, and 21 (1%) chose another gender option. The mean age was *M* = 42 (SD = 15) and 67% of participants had at least a bachelor’s degree.

### Measures

As the two main outcome measures across all studies, participants were asked to rate the author competence on three items (e.g., “The author is knowledgeable of the subject.”; Cronbach’s *α*_Study 1_ = 0.90, *α*_Study 2a_ = 0.91, *α*_Study 2b_ = 0.91, *α*_Study 3_ = 0.89) as well as the content of the recommendation on five items (e.g., “The text is credible.”; Cronbach’s *α*_Study 1_ = 0.80, *α*_Study 2a_ = 0.77, *α*_Study 2b_ = 0.75, *α*_Study 3_ = 0.86). Given the good internal consistency of these multi-item measures, as preregistered, we used the mean values in all analyses. Furthermore, in Studies 1, 2a and 2b we further assessed the participants’ behavioral intention to share the text with friends and family with one item. Only in Studies 2a and 2b participants were further asked about their intention to follow the recommendations with three items (e.g., “I intend to follow the provided recommendations.”; Cronbach’s *α*_Study 2a_ = 0.92, *α*_Study 2b_ = 0.95). Responses to all items across measures were given on a 7-point Likert-type response scale ranging from “strongly disagree” to “strongly agree.” In Study 3, the main outcome measure was participants’ choice from whom they would like to receive advice for a randomly selected societal challenge (binary decision between a human expert vs. generative AI).

Additionally, participants answered some additional questions after the main outcome measures: who they thought the author of the text was (AI or human) on a 7-point Likert-type scale ranging from “definitely a human” to “definitely an artificial intelligence (AI)” (only in the unknown author condition of Study 1); their subjective knowledge about the context on a 7-point Likert-type scale ranging from “no knowledge at all” to “extremely much knowledge” (only in Study 1); how relevant the topic is for them personally on a 7-point Likert-type scale ranging from “not relevant at all” to “extremely relevant” (only in Studies 2a and 2b); as well as about their age, gender, and education (in all studies).

### Procedure

The studies were conducted online in the order as they are reported here. Participants were recruited via Prolific (https://www.prolific.com/); participants from previous studies were not invited to any of the following studies. The order of the outcome measures was randomized in Study 1, Study 2a, and Study 2b. In Study 3, participants first evaluated the content of the recommendations in the experience trial, followed by their choice from whom they would like to receive advice for a randomly selected societal challenge (but without learning about the specific advice), and their evaluation of both authors’ competence. Importantly, in Study 3 and in the transparent conditions of the other studies, participants evaluated the content without knowing the identity of the author.

### Generative AI content

We used ChatGPT-3.5 to generate AI content. For the introductory paragraph, we used the following prompt: “Write an introductory paragraph for a scientific paper dealing with how artificial intelligence can help to provide solutions to societal challenges (e.g., climate change, pandemic preparedness) and personal challenges (e.g., improving healthy eating, saving money). Do not include examples on predictive AI or on how AI can analyze data. Do not include references to scientific papers.”

In Studies 1 and 3, we used ChatGPT-3.5 to generate responses to questions concerning various societal challenges. We selected questions and responses from interviews with human experts from science and practice in which they answered a rather broad question regarding a specific societal challenge. The societal challenges were global warming, pandemic preparedness, refugee reception, fake news (only Study 1), and effect of AI on the workforce (only Study 1). These specific contexts were selected based on their identification as pressing challenges by global institutions such as the European Union or the United Nations, as well as the availability of corresponding expert recommendations that were also suited as prompts for generative AI. For instance, for the societal challenge of pandemic preparedness, the question to be answered was “What are some of the biggest challenges in increasing pandemic preparedness?”; answered by Richard Hatchett, CEO of the Coalition for Epidemic Preparedness Innovations, in an interview with McKinsey from October 2021. ChatGPT was prompted with the exact same question. If the initial AI response was either much longer or shorter than the response from the human expert, we asked the AI to shorten or extend its response, respectively. All the questions and the respective responses by human experts and ChatGPT are provided in the “Stimuli” section in the Supplementary Information.

In Studies 2a and 2b, we used ChatGPT-3.5 to generate responses to questions concerning various personal challenges The contexts included in this study—exercising more regularly, quitting smoking, eating healthier, reducing time spent on the phone, saving money, having a positive impact on the world—were chosen based on the authors’ internal discussion, aiming to include personal challenges that would be relevant for many individuals from the target population. For example, ChatGPT was prompted with “How can I save more money?” (see the “Stimuli” section in the Supplementary Information for all prompts and responses).

### Statistics and reproducibility

The hypotheses and analyses were preregistered on 2023-01-04 for Study 1 (https://aspredicted.org/xj2yn.pdf), on 2023-01-14 for Study 2a (https://aspredicted.org/h9y6v.pdf), on 2023-04-07 for Study 2b (https://aspredicted.org/w86qh.pdf), and on 2023-06-29 for Study 3 (https://aspredicted.org/kp2sb.pdf). Data distribution of continuous outcome measures was assumed to be normal, but this was only visually not formally tested (see the Supplementary Information: Supplementary Figs. 3–6 for Study 1, Supplementary Figs. 10–17 for Studies 2a and 2b, Supplementary Fig. 18 for Study 3). We conducted all confirmatory analyses as preregistered. Additional analyses are indicated as exploratory. All full models of analyses reported here and additional robustness checks with demographic controls are reported in the “Supplementary Tables and Figures” section in the Supplementary Information. We report two-tailed *p*-values. Bonferroni-corrected *p*-values are referred to as *p*_BC_.

Null findings reported in this paper are accompanied by equivalence tests examining the hypothesis that the effect size *η*^2^ in the ANOVA model is greater than or at least as great as our smallest effect size of interest (SEOI) *∆* = 0.01, corresponding to a small effect based on standard conventions. If the equivalence test proves to be statistically significant (e.g., *p*_eq_ < 0.05), we conclude that the effect is practically equivalent and that the data are most compatible with no important effect because *η*^2^ is likely to be smaller than *∆*. In contrast, if *p*_eq_ ≥ 0.05, we consider the observed evidence to be inconclusive^22^.

### Reporting summary

Further information on research design is available in the Nature Portfolio Reporting Summary linked to this article.

## Results

### Study 1

The goal of Study 1 was to compare people’s evaluation of potential solutions to pressing societal challenges as recommended either by human experts or generative AI (i.e., ChatGPT). Following the algorithm aversion account, we hypothesized that (i) recommendations by AI (vs. human experts) to solve societal challenges as well as (ii) the author of such recommendations would be devaluated and (iii) shared less likely with others when the author identity is (vs. is not) made transparent.

Our focus is on examining whether making the author identity transparent—disclosing whether the recommendation was generated by an AI or a human expert—affects the focal outcome variables. To this end, we are interested, as preregistered, in the interaction effect between author identity and author transparency. In a first step, we conducted a MANOVA with the three experimental factors as independent variables (i.e., author identity, author transparency, and context), and all three outcomes as dependent variables (i.e., author competence, content quality, and sharing intention). Results indicate a main effect of author identity, Pillais’ Trace = 0.05, *F*(3, 981) = 17.53, *p* < 0.001, author transparency, Pillais’ Trace = 0.01, *F*(3, 981) = 4.16, *p* = 0.006, and context, Pillais’ Trace = 0.02, *F*(12, 2949) = 1.94, *p* = 0.026. Additionally, there was a significant interaction effect between author identity and author transparency, Pillais’ Trace = 0.05, *F*(3, 981) = 8.53, *p* < 0.001.

**Fig. 1:**
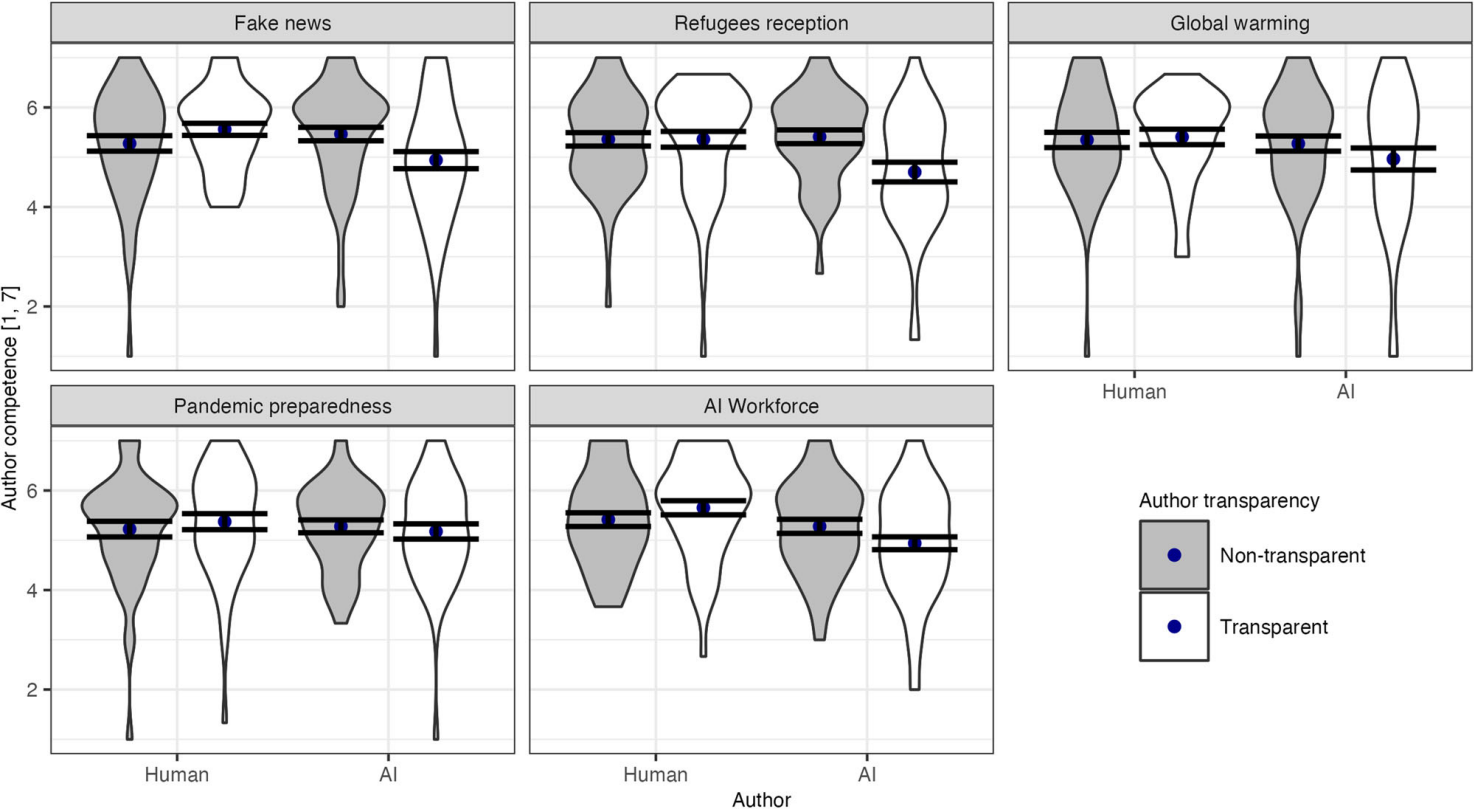
Perceived author competence across contexts by author transparency in Study 1 (*N* = 1003). Author competence was calculated as the mean value from three response items (see the “Methods” section). Dots indicate mean values, error bars represent 95% confidence intervals, and gray/white areas display kernel densities. Similar figures for the other outcome variables are available as Supplementary Figs. 1 and 2 in the Supplementary Information.

To test our hypotheses, we subsequently conducted ANOVAs, separately for each of the three outcome measures. Regarding, perceived author competence, we indeed found a significant interaction effect between author identity and author transparency, *F*(1, 983) = 15.84, *p*_BC_ = 0.001, Cohen’s *f* = 0.13. This effect was robust across the five contexts studied (three-way interaction including context: *F*(4, 983) = 0.55, *p*_BC_ = .999, *p*_eq_ = 0.009, Cohen’s *f* = 0.05), as well as when adding demographic controls (see Supplementary Table 1 in the Supplementary Information). As shown in Fig. 1, the perceived competence of AI authors (*M* = 5.35, SD = 1.01) was similar to those of human authors (*M* = 5.32, SD = 1.05) when the author identity was non-transparent (as also indicated by a non-significant effect of author identity in the non-transparent condition alone, *F*(1, 498) = 0.06, *p*_BC_ = 0.999, *p*_eq_ = 0.016, Cohen’s *f* = 0.05). This resonates with the exploratory finding that in the non-transparent condition, participants who read an AI-generated recommendation (*M* = 3.27, SD = 1.36) or a human-generated recommendation (*M* = 3.32, SD = 1.42) did not statistically differ in their average estimation of whether the text was written by an AI or human author, *F*(1, 506) = 0.19, *p*_BC_ = 0.665, *p*_eq_ = 0.030, Cohen’s *f* = 0.02 (see also^4^). Adding participants’ subjective knowledge about the context as an additional predictor did not affect this result qualitatively (see Supplementary Table 2 in the Supplementary Information). In contrast, AI authors were rated as less competent (*M* = 4.95, SD = 1.22) relative to human authors (*M* = 5.47, SD = 1.02) when their identity was transparent (effect of author identity in the transparent condition alone: *F*(1, 485) = 25.92, *p*_BC_ < 0.001, Cohen’s *f* = 0.23).

Repeating the same analyses for content evaluations and sharing intentions, in contrast, the focal interaction effect between author identity and author transparency was not statistically significant, both for content evaluation, *F*(1, 983) = 0.80, *p*_BC_ = 0.999, *p*_eq_ = 0.012, Cohen’s *f* = 0.03, and for sharing intentions, *F*(1, 983) = 0.07, *p*_BC_ = 0.999, *p*_eq_ = 0.002, Cohen’s *f* = 0.01. Based on the equivalence tests for these effects, we reject the presence of a meaningful effect.

Taken together, these results indicate that although people exhibit AI aversion in terms of perceived author competence, this aversion does not extend to their evaluation of the content quality or their sharing intentions. This finding suggests that people generally like recommendations to address pressing societal challenges generated by AI as much as those generated by human experts, although they clearly devaluate the competence of AI advisors.

To explore whether the observed interaction effect between author identity and author transparency on perceived author competence was due to a general aversion to AI authors or rather a preference for human authors, we examined whether author transparency had a differential effect on author competence in the AI and human author conditions. We found that in the case of an AI author, transparency (*M* = 4.95, SD = 1.22) decreased perceived author competence compared to non-transparency (*M* = 5.35, SD = 1.01), *F*(1, 507) = 15.61, *p*_BC_ < 0.001, Cohen’s *f* = 0.18. In the case of a human author, transparency (*M* = 5.47, SD = 1.02) did not result in statistically different competence ratings compared to non-transparency (*M* = 5.32, SD = 1.05), *F*(1, 476) = 2.52, *p*_BC_ = 0.113, *p*_eq_ = 0.271, Cohen’s *f* = 0.07 (although we cannot reject the presence of a small effect based on the equivalence test). Thus, the observed interaction effect on author competence is more likely to be the result of AI aversion than of human appreciation under author transparency.

### Study 2a

The goal of Study 2a was to replicate the effects of Study 1 in the context of personal challenges. With this, we aimed to increase the personal relevance of the context. Extending the previous study, we added another outcome measure to investigate the potential spillover effect of a (negative) author evaluation to participants’ intention to follow the advice. We hypothesized that an AI author’s competence would be devaluated, and the recommendation would be less likely followed when its identity is transparent (vs. non-transparent); following the results of Study 1, we did not hypothesize differences regarding author transparency in the content evaluation and sharing intention.

As intended, participants reported on average a high personal relevance of the self-selected personal challenge (*M* = 6.42, SD = 0.73). The share of participants opting for either of the contexts was as follows: 20% exercising more, 6% quitting smoking, 18% eating healthier, 9% reducing screen time, 35% saving money, and 12% having a positive impact on the world.

**Fig. 2:**
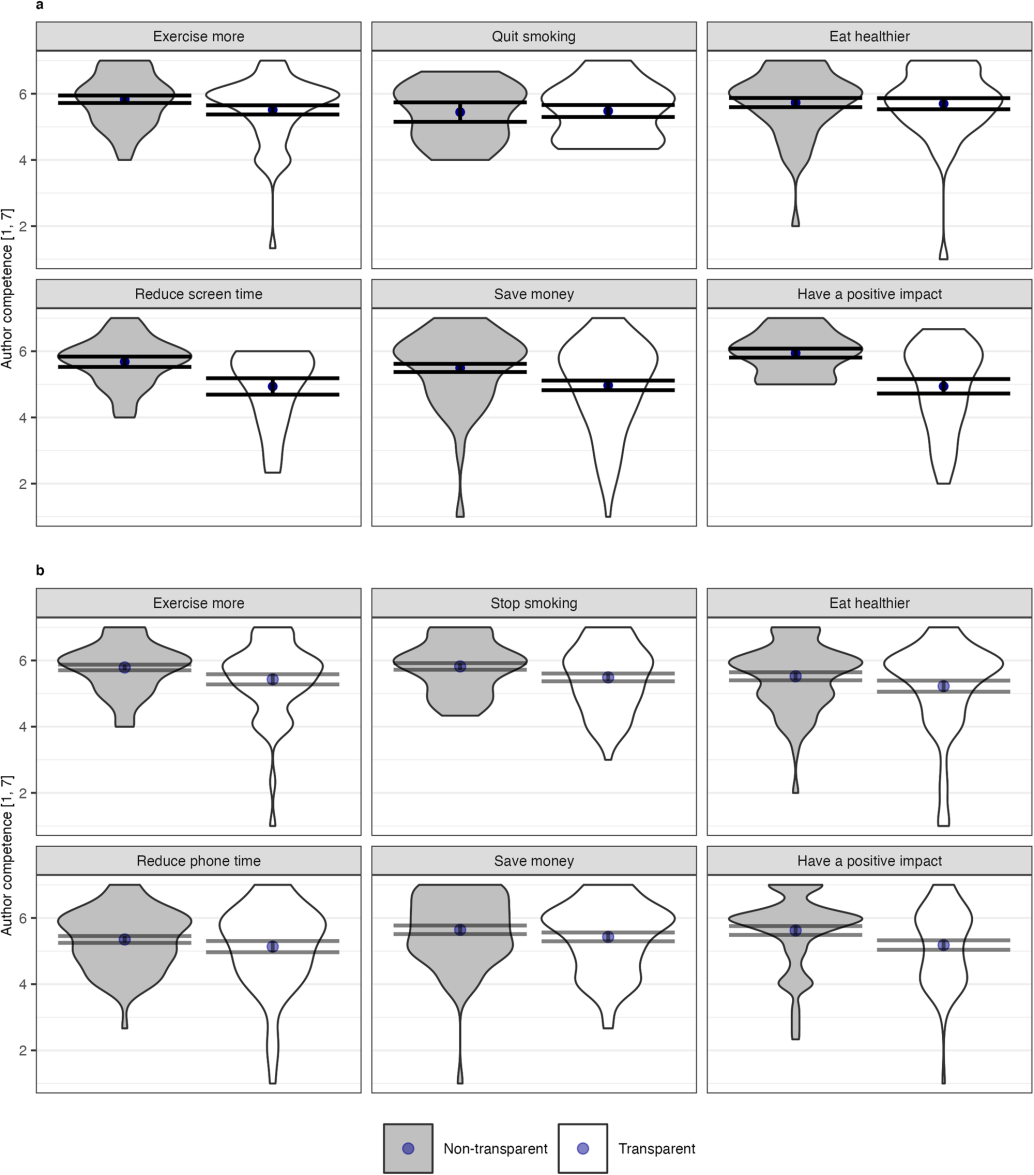
Perceived author competence across contexts by author transparency. Studies 2a (panel **a** *N* = 501) and 2b (panel **b** *N* = 800). Author competence was calculated as the mean value from three response items (see the “Methods” section). Dots indicate mean values, error bars represent 95% confidence intervals, and gray/white areas display kernel densities. Similar figures for the other outcome variables are available as Supplementary Figs. 7–9 in the Supplementary Information.

To test our hypotheses, we conducted an ANOVA on perceived author competence with author transparency as the independent variable. As hypothesized, we again found that the AI author was perceived as less competent when its identity was transparent (*M* = 5.24, SD = 1.21) than when it was non-transparent (*M* = 5.67, SD = 0.97), *F*(1, 489) = 20.04, *p* < 0.001, Cohen’s *f* = 0.20. The effect does not change qualitatively when adding demographic controls (see Supplementary Table 3 in the Supplementary Information). Despite some descriptive differences across contexts as shown in Fig. 2a, the author transparency × context interaction effect did not reach statistical significance, *F*(5, 489) = 1.93, *p* = 0.087, *p*_eq_ = 0.541, Cohen’s *f* = 0.14. However, given that we cannot reject a small effect based on the equivalence test, we refrain from speculations whether the differences between contexts are due to random variation or are subject to systematic differences related to the nature of the contexts.

Further replicating the results of Study 1, there were no effects of author transparency on content evaluation, *F*(1, 489) = 0.1, *p* = 0.752, *p*_eq_ = 0.023, Cohen’s *f* = 0.01, and sharing intention, *F*(1, 489) = 0.96, *p* = 0.329, *p*_eq_ = 0.105, Cohen’s *f* = 0.04. Moreover, author transparency did also not affect the newly added measure of perceived likelihood to follow the recommendation, contrary to our hypothesis, *F*(1, 489) = 0.54, *p* = 0.462, *p*_eq_ = 0.066, Cohen’s *f* = 0.03. Based on additional equivalence tests, we can reject a small effect for content evaluation, whereas the results are inclusive for sharing intention as well as for perceived likelihood to follow the recommendation.

Taken together, we replicated the finding that making the identity of an AI author transparent leads to a devaluation of the author competence, but this does not appear to meaningfully reduce the evaluation of and behavioral intention to follow or share the recommendation.

### Study 2b

The goal of Study 2b was to replicate and extend the findings from Study 2a. Specifically, we re-run the same study design with one important difference: In Study 2a, participants self-selected one context by stating which personal challenge was most relevant to them personally. Our reasoning was that when people choose a context personally relevant to them, they are likely more motivated to accurately evaluate the stimulus materials. However, self-selection also creates the problem of endogeneity bias. Therefore, we aimed to replicate Study 2a while using a random allocation of participants to the different contexts.

As expected from the random assignment to contexts, participants reported on average a descriptively lower level of personal relevance of the topic (*M* = 5.18, SD = 1.44) compared to Study 2a. To test our hypothesis, we conducted an ANOVA on perceived author competence with author transparency as the independent variable. As expected and replicating the results from Study 2a, the AI author was perceived as less competent when its identity was transparent (*M* = 5.32, SD = 1.17) than when it was non-transparent (*M* = 5.62, SD = 0.95), *F*(1, 788) = 16.46, *p* < 0.001, Cohen’s *f* = 0.15. As shown in Fig. 2b, the effect was descriptively present across all six contexts, as also evident by a statistically non-significant author transparency × context interaction effect, *F*(5, 788) = 0.23, *p* = 0.953, *p*_eq_ = 0.002, Cohen’s *f* = 0.04, the absence of a meaningful effect was further supported by an equivalence effect. Repeating this analysis in an OLS framework with the continuous level of personal relevance as an additional predictor variable, we replicated the effect of author transparency, *B* = −0.30, SE = 0.08, *p* < 0.001, 95% CI [−0.46, −0.14], and also found a positive effect of personal relevance, *B* = 0.17 SE = 0.04, *p* < 0.001, 95% CI [0.10, 0.25] (the interaction effect was not significant for the null-hypothesis test, *B* = 0.07 SE = 0.06, *p* = 0.205, *p*_eq_ = 0.124, 95% CI [−0.04, 0.19], although the additional equivalence test yields the finding inconclusive). This indicates that the author was evaluated as more competent when the participants perceived a greater personal relevance of the described challenge.

Additionally, we explored the effects on the other outcome measures. In line with the previous studies, there were no significant effects of author transparency on any of the measures, what was further supported by additional equivalence tests in case of content evaluation, *F*(1, 788) = 1.2, *p* = 0.273*, p*_eq_ = 0.042, Cohen’s *f* = 0.04, and sharing intention, *F*(1, 788) = 0.85, *p* = 0.356, *p*_eq_ = 0.029, Cohen’s *f* = 0.03, but inconclusive for the likelihood to follow the recommendation, *F*(1, 788) = 2.56, *p* = 0.110, *p*_eq_ = 0.110, Cohen’s *f* = 0.06. The interpretations remain when adding demographic controls to the statistical models (see Supplementary Tables 4–7 in the Supplementary Information).

Taken together, the study replicates the results from Study 2a. That is, after reading recommendations on how to address personal challenges, the author of the recommendation receives lower competence ratings when its identity as an AI agent is known (vs. unknown). This effect appears to be independent of whether the evaluators self-select a context of personal relevance (Study 2a) or when they are assigned randomly to a context (Study 2b).

### Study 3

The previous studies indicate that advisors providing recommendations on how to address societal or personal challenges are evaluated as less competent when they are identified as AI agents. While being interesting from a theoretical perspective, however, one may argue that this effect is largely irrelevant given that we found no evidence that the recommendations knowingly coming from AI agents are evaluated (more) negatively. Therefore, the aim of Study 3 was to investigate a potential consequence of lower levels of competence attributed to AI versus human advisors. To this end, we examined whether people are more likely to choose human advice for addressing societal challenges than AI advice. We reasoned that identifiable prior experience with advice for another societal challenge and, thus, learning about the quality of AI- vs. human-generated advice, could increase the preference for receiving AI advice. Accordingly, the study varied whether people learned (vs. learned not) about the author identity of previous advice by AI and human authors prior to the choice task. We hypothesized that (i) a potential bias in selecting human advisors more likely than AI advisors would be weaker when people gained transparent prior experience with human and AI advice, (ii) particularly when they evaluated the prior AI-generated advice as more positive relative to the human-generated advice.

In a first step, we analyzed how people evaluated the recommendations they received in the experience stage. Results from a mixed effects ANOVA indicate that AI-generated advice (*M* = 5.42, SD = 0.92) was evaluated more positively than the human-generated advice (*M* = 5.01, SD = 1.23), *F*(1, 1000) = 112.37, *p* < 0.001, Cohen’s *f* = 0.34.

**Fig. 3:**
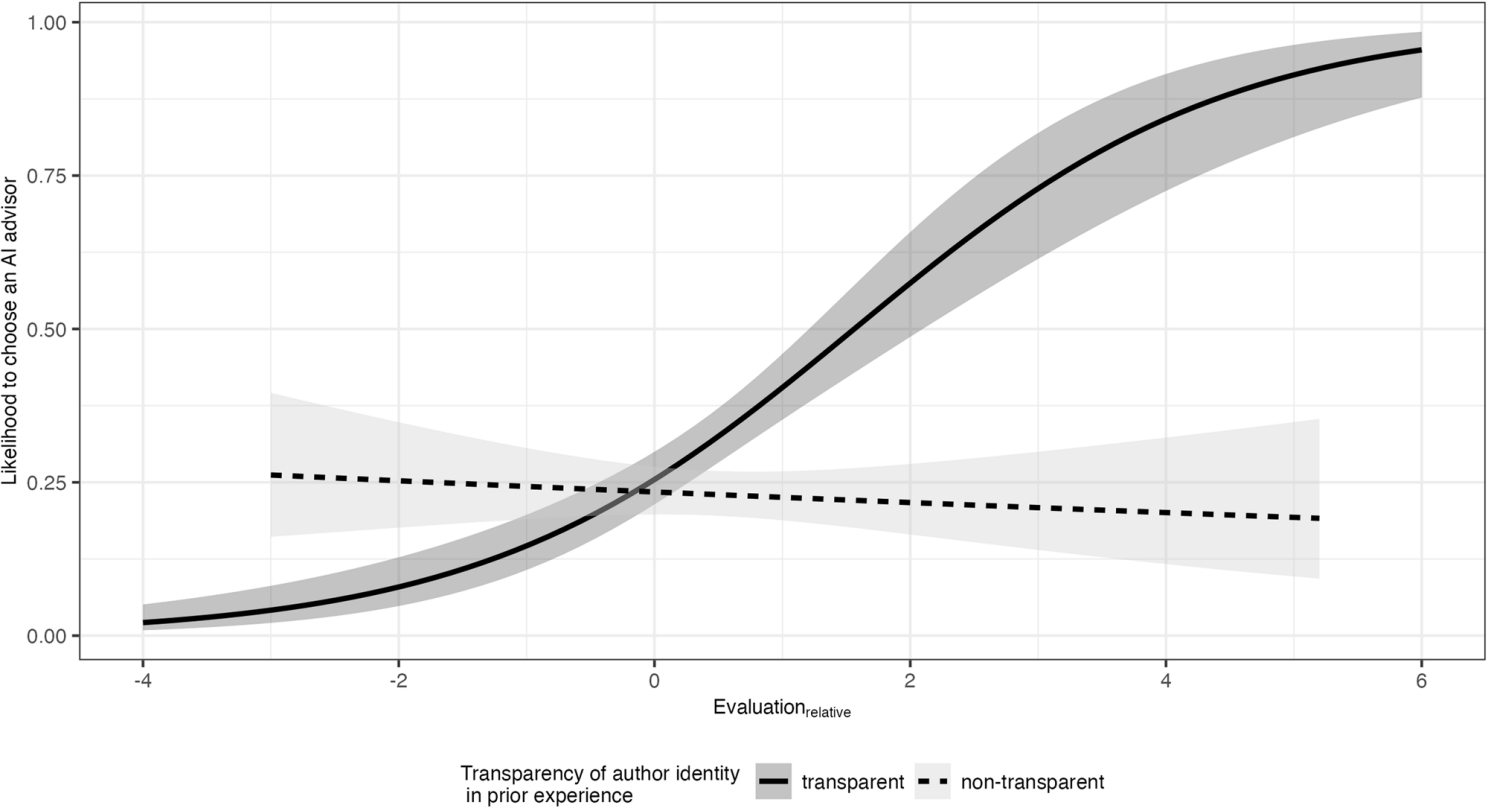
Likelihood to choose an AI advisor for a societal challenge, depending on transparency of author identity in prior experience and on the relative evaluation or prior experience (*N* = 1004). Choice for AI advisor (coded as 1) compared to a human advisor (coded as 0). Evaluation_relative_ = content evaluation_AI_ – content evaluation_human_. The gray areas display the 95% confidence intervals.

To test our first hypothesis, we next conducted a logistic regression on the choice for a human ( = 0) vs. AI ( = 1) advisor by transparency of author identity in prior experience and context. As expected, the choice for the AI author was about 1.6 times more likely when the author identity in the experience stage was transparent (32%) than when it was non-transparent (23%), *B* = −0.46., SE = 0.14, *p* = 0.001, inverse OR = 1.59, 95% CI [−0.75, −0.18]. However, this effect might be driven by the fact that the AI-generated advice was evaluated more positively than the human-generated advice in the experience stage. Therefore, as preregistered, we computed the algebraic difference of content evaluation_relative_ = content evaluation_AI_ – content evaluation_human_, with positive values indicating a more favorable evaluation of the AI- vs. the human-generated content and negative values vice versa. This difference score was used as an additional predictor in the logistic regression. As hypothesized, we found a significant interaction effect between transparency of author identity in prior experience and content evaluation_relative_, *B* = −0.73, SE = 0.13, *p* < 0.001, inverse OR = 2.07, 95% CI [−1.00, −0.47]. As shown in Fig. 3, when the author identity was transparent, participants’ choice for the AI advisor increased the more positive their prior experience with the AI vs. human advisor was, *B* = 0.69, SE = 0.10, *p* < 0.001, OR = 1.99, 95% CI [0.50, 0.89]. When the author identity was non-transparent, in contrast, the relative evaluation of the previously received content did not affect the choice, *B* = −0.05, SE = 0.09, *p* = 0.567, inverse OR = 1.05, 95% CI [−0.22, 0.12]. Interestingly, the intersection of the two slopes is close to zero (see Fig. 3), indicating that only when participants evaluated the AI-generated content more positively than the human-generated content, they would increase the likelihood to choose an AI advisor compared to a situation with no transparent experience.

Taken together, Study 3 extends our results from the previous studies by pointing to a potential consequence of a lower perceived competence of AI authors compared to human authors. That is, people prefer to choose human over AI advisors. Importantly, however, when people gain positive experience with AI-generated content, their willingness to choose AI-generated advice increases.

## Discussion

Recent advancements in artificial intelligence have the potential to offer valuable insights in relevant societal and personal issues. That is, generative AI may provide ideas and nudges on how to address such challenges even for non-experts. Yet, the effectiveness of AI-generated advice also depends on how people evaluate it and whether AI is approached in the first place for advice. Our findings indicate that based on the given evidence, people perceive AI advisors to be less competent than human (expert) advisors when the identity of the advisor is revealed, as opposed to being anonymous. The results additionally suggest that this difference in perceived author competence is primarily driven by a devaluation of AI rather than an appreciation of human authorship^19^. It is important to note that this devaluation of AI competence is not associated with a devaluation of the AI-generated advice itself or a decreased willingness to implement or share it. Nevertheless, AI (vs. humans) are chosen less likely as advisors in addressing societal and personal challenges. Importantly, however, our findings propose that positive experience with AI-generated advice mitigates such AI aversion.

The results are noteworthy in light of previous research suggesting that people believe algorithms are incapable of effectively performing tasks that require subjectivity^17^. This aspect is closely related to addressing societal and personal challenges, for which an “objectively correct” solution does oftentimes not exist. One notable distinction between generative AI such as ChatGPT, which was employed to generate advice in the present studies, and other artificial recommendation systems is that ChatGPT’s recommendations are presented in a clear and easily understandable manner, potentially reducing the overall skepticism towards recommendations given by AI/algorithms^23^. This may be attributed to the reduced perceived complexity of the recommendations^9^.

A possible consequence of increased use of generative AI systems by laypeople is that it may have positive effects on the acceptance of AI more generally. Our findings suggest that positive experiences with AI-generated advice can decrease AI aversion and increase individuals’ preference for AI advisors. Providing opportunities for individuals to interact with and learn about AI—through educational programs, demonstrations, or hands-on experiences—could therefore be an effective way to increase acceptance and adoption of AI as an advisor.

Our findings remain consistent in contexts of high personal relevance to participants, specifically in the context of personal challenges. This result contributes to recent discussions on whether individuals may exhibit greater aversion toward algorithms in situations that involve elements of personal identity^19^. We propose that personal identity relevance may be more prominent in situations that involve personal encounters, whereas the recommendations provided by generative AI in addressing societal or personal challenges may not elicit a strong sense of interaction. Further research is needed to identify the common and distinct characteristics of human-AI/algorithm interactions, as well as to investigate the circumstances under which AI/algorithm aversion emerges and its impact on outcomes. In any case, the availability of easy to access and easy to use generative AI such as ChatGPT adds a new dimension to the ongoing discourse on this topic.

Although AI-generated advice was not found to be devalued per se, the fact that AI authors were perceived as less competent than human authors when the author identity was known has important implications that can be tested in future research. For instance, some work suggests that higher levels of perceived capability can lead to higher expectations regarding outcomes^24^. Higher expectations, in turn, could lead to more blaming when the outcome does not meet the expectations. Thus, it might be fruitful to investigate the relation between the perceived competence of generative AI, evaluation of the content generated by AI, and blame (oneself vs. AI advisor) for negative outcomes following the content evaluation by others. Other research found that individuals cannot recognize if a conversation mixes human- and AI-generated elements^25^, which becomes more relevant with the increasing exposure and use of AI-based chatbots and writing assistants. Thus, future research could investigate whether it is better to explicitly mark human- and AI-generated content to prevent human content to be devaluated or whether it is better to not mark the source as AI content might profit from the more positive evaluation of the human author.

### Limitations

It is important to acknowledge the limitations of our research. Firstly, while we examined a variety of contexts related to societal and personal challenges, we naturally cannot claim generalizability of our findings to other contexts. Secondly, we made efforts to ensure equivalent expert/competence framing across the AI and human conditions in Study 1, as differences in framing may be a potential source of algorithm aversion^26^. However, it is possible that even slight variations in framing may have influenced our results. Relatedly, there may also be objective differences in the quality of the content generated by AI- vs. human-generated content. Although we found no clear pattern of evidence regarding whether the content generated by AI vs. human authors is evaluated differently, this is of course only a snapshot of the specific contexts, prompts, and generative AI models used in these studies. It is important to note, however, that our main findings are based on comparisons between transparent versus non-transparent author identity within the AI author condition (in fact, Studies 2a and 2b did not even include human-generated content), which employed consistent content and framing throughout. Finally, we employed different measures of author and content evaluation that were tailored to the respective context, which may have led to variations in responses. However, our crucial comparison relies on the same outcome measure, that is, author evaluation when the author was transparent versus non-transparent.

## Conclusions

Overall, our work contributes to the understanding of AI/algorithm aversion and proposes a more nuanced perspective. Specifically, our findings indicate that people are not necessarily averse towards the advice generated by AI but rather toward the AI advisor itself. We also add to the AI aversion literature by demonstrating that positive experiences with generative AI can reduce AI aversion, a finding that may help reconcile conflicting views on AI aversion in the existing literature. More broadly, our findings present a more optimistic outlook regarding the potential receptivity and implementation of the new generation of generative AI tools, compared to what has been previously suggested. In conclusion, generative AI’s clear and easily understandable recommendations could indeed appear helpful to human decision makers to address various societal and personal challenges.

### Supplementary information

Supplementary Information

Reporting Summary

Peer Review File

## Data Availability

The study materials and data are available on the Open Science Framework at https://osf.io/6ptx8/.

## Code availability

The code used for analyzing the data is available on the Open Science Framework at https://osf.io/6ptx8/. All analyses were conducted with R (version 4.2.3).
