# Supplementary material for: People devalue generative AI’s competence but not its advice in addressing societal and personal challenges
Source: Commun Psychol. 2023 Nov 15;1:32. doi: 10.1038/s44271-023-00032-x (PMC11332189; doi:10.1038/s44271-023-00032-x)

Supplementary Information for: People devalue generative AI's competence but not its advice in addressing societal and personal challenges

Robert Böhm[1,2,*], Moritz Jörling[3], Leonhard Reiter[1], Christoph Fuchs[4]

[1] Faculty of Psychology, University of Vienna, Universitätsstrasse 7, 1010 Vienna, Austria

[2] Department of Psychology and Copenhagen Center for Social Data Science (SODAS), University of Copenhagen, Øster Farimagsgade 2a, 1353 Copenhagen K, Denmark

[3] Marketing Department, EMLyon Business School, 23 Av. Guy de Collongue, 69130 Écully, France

[4] Faculty of Business, Economics, and Statistics, University of Vienna, Oskar-Morgenstern-Platz 1, 1090 Vienna, Austria

* Corresponding author: robert.boehm@univie.ac.at

Content

Supplementary Methods *2*
Preregistrations *2*
Study 1 .... *2*
Study 2a *2*
Study 2b *2*
Study 3 .... *2*
Stimuli *2*
Studies 1 and 3 .... *2*
Studies 2a and 2b *14*
Supplementary Tables and Figures *27*
Study 1 .... *27*
Studies 2a and 2b *34*
Study 3 .... *48*

Supplementary Methods

All data, analysis code and surveys are available here: https://osf.io/6ptx8/

Preregistrations

Study 1

Available at https://aspredicted.org/xj2yn.pdf

Study 2a

Available at https://aspredicted.org/h9y6v.pdf

Study 2b

Available at https://aspredicted.org/w86qh.pdf

Study 3

Available at https://aspredicted.org/kp2sb.pdf

Stimuli

Studies 1 and 3

What do you think social media platforms should do to better tackle fake news?

From: How can social media platforms better prevent fake news? 3 questions to Evelyn Douek

Evelyn Douek (lecturer on law and S.J.D. candidate at Harvard Law School, and Affiliate at the Berkman Klein Center For Internet & Society. She studies global regulation of online speech, private content moderation institutional design and comparative free speech law and theory)

This is a huge question! Thousands of trees have been felled by regulators and academics around the globe writing reports and articles trying to answer this question, so in my small space here let me do a classic academic move and break down the question into smaller questions instead.

First, ‘fake news’ is a nebulous term that means different things to different people, and has been co-opted by a number of actors to just simply mean ‘news I don’t like’. So it’s useful to be a little more precise about the different types of content that may require intervention by platforms. Wardle and Derakhshan developed a helpful and widely-cited taxonomy breaking it down into three categories: disinformation (false information spread with intention to harm), misinformation (false but not intended to cause harm), and mal-information (information based on reality and intended to cause harm). Even within these there will be subcategories depending on the actor behind the content and the methods they use to try to spread it. For example, you might deal with

false clickbait articles being spread by people trying to make a profit differently to a well-resourced state-backed information operation targeted at a specific electoral process. Sorry to take your question about a single hard problem and turn it into multiple hard problems, but it's no wonder platforms and regulators are struggling to find the right approach! It's hard!

Even though there's no single solution, as an overarching observation I would just say that we need to avoid overly simplistic or knee-jerk responses. Requiring platforms to adjudicate whether all the content on their platforms is true or not and take down anything deemed 'false' is not the right path: it's not practically possible, it will endanger legitimate freedom of expression, and it's not clear that simply removing content is necessarily the best way of correcting people's false beliefs.

A better approach is to get out of the take-down/leave-up paradigm of content moderation and leverage the full variety of options that platforms have available to them. For example, they can add labels to content showing if it's been fact-checked or manipulated, provide additional context or transparency about particular posts or accounts, add friction so that content doesn't spread as far or as fast (such as by downranking content in the feed or changing platform affordances like how easy it is to share content), highlight authoritative information, help make counter-messaging easier or more effective, and many many more similar kinds of interventions. All of this will require imagination, experimentation and independent empirical studies to assess the effectiveness of various interventions, but I believe they will ultimately be more fruitful than relying on blunt tools.

ChatGPT content

Social media platforms should consider implementing stronger verification processes for accounts and news sources in order to ensure that only credible sources are able to disseminate information on the platform. This could involve verifying the identity of accounts, as well as verifying the credibility and reliability of news sources. In addition, social media platforms should provide more accurate and transparent information about the sources of news to help users evaluate the credibility of the information they are seeing and make informed decisions about what to believe. This could involve clearly labeling news sources and providing information about their credibility, as well as providing context about the news story and its sources.

To help users identify and report fake news, social media platforms can offer tools and resources such as fact-checking tools and clear guidelines for reporting fake news and misinformation. These tools can help users to identify false or misleading information and take action to report it to the platform. Additionally, promoting media literacy can be an effective strategy for helping users to identify fake news and misinformation. This could involve providing tips and resources

for evaluating the credibility of news sources and spotting fake news, as well as promoting media literacy education more broadly.

Finally, social media platforms can collaborate with fact-checking organizations to identify and label false or misleading content, and to remove it from the platform. This can help to ensure that false or misleading information is identified and removed as quickly as possible. Overall, it's important for social media platforms to take a proactive and comprehensive approach to tackling fake news, which will likely involve a combination of the strategies mentioned above, as well as continued innovation and experimentation to identify and implement effective solutions.

ChatGPT screenshot

What do you think social media platforms should do to better tackle fake news? Long answer without bullet points

Social media platforms should consider implementing stronger verification processes for accounts and news sources in order to ensure that only credible sources are able to disseminate information on the platform. This could involve verifying the identity of accounts, as well as verifying the credibility and reliability of news sources. In addition, social media platforms should provide more accurate and transparent information about the sources of news to help users evaluate the credibility of the information they are seeing and make informed decisions about what to believe. This could involve clearly labeling news sources and providing information about their credibility, as well as providing context about the news story and its sources.

To help users identify and report fake news, social media platforms can offer tools and resources such as fact-checking tools and clear guidelines for reporting fake news and misinformation. These tools can help users to identify false or misleading information and take action to report it to the platform. Additionally, promoting media literacy can be an effective strategy for helping users to identify fake news and misinformation. This could involve providing tips and resources for evaluating the credibility of news sources and spotting fake news, as well as promoting media literacy education more broadly.

Finally, social media platforms can collaborate with fact-checking organizations to identify and label false or misleading content, and to remove it from the platform. This can help to ensure that false or misleading information is identified and removed as quickly as possible. Overall, it's important for social media platforms to take a proactive and comprehensive approach to tackling fake news, which will likely involve a combination of the strategies mentioned above, as well as continued innovation and experimentation to identify and implement effective solutions.

What should be the role of local authorities and other levels of government in migrant and refugee reception?

From: Local challenges and solutions for migration: an interview with Bastien Revel - The Hague Academy

Bastien Revel (independent consultant with over 13 years of peacebuilding and governance experience with various international organisations and NGO's)

Local authorities play a key role in organising migrants' and refugees' very first steps in their new communities — for example, by finding accommodation and shelter. It is also crucial that the host community — which can be overwhelmed by a sudden influx — sees that their representatives are involved in the process. Experience in Istanbul shows that early efforts to prevent refugees or migrants from concentrating in the same street or neighbourhood helps them integrate. It is also important for local authorities to keep track of refugees and migrants living in their area.

Cooperation with other levels of government is very important, as a good deal of registration data is held centrally. Other levels of government establish the framework for refugees' access to services.

ChatGPT content

The role of local authorities and other levels of government in migrant and refugee reception involves providing access to basic services such as healthcare, education, and housing, promoting cultural understanding and awareness within the community, facilitating economic integration through job training and employment opportunities, and coordinating with other organizations and stakeholders to ensure that migrants and refugees receive the support they need. It's important for local authorities and other levels of government to work collaboratively with migrants and refugees, as well as with other organizations and stakeholders, to ensure that the needs of migrants and refugees are met and that they are able to successfully integrate into their new communities.

ChatGPT screenshot

What should be the role of local authorities and other levels of government in migrant and refugee reception? Short answer without bullet points

The role of local authorities and other levels of government in migrant and refugee reception involves providing access to basic services such as healthcare, education, and housing, promoting cultural understanding and awareness within the community, facilitating economic integration through job training and employment opportunities, and coordinating with other organizations and stakeholders to ensure that migrants and refugees receive the support they need. It's important for local authorities and other levels of government to work collaboratively with migrants and refugees, as well as with other organizations and stakeholders, to ensure that the needs of migrants and refugees are met and that they are able to successfully integrate into their new communities.

What are some of the biggest challenges in increasing pandemic preparedness?

From: Accelerating pandemic response efforts: An interview with CEPI's Richard Hatchett | McKinsey

Richard Hatchett (CEO of the Coalition for Epidemic Preparedness Innovations)

The biggest challenges are around vaccine manufacturing—and these are manifold. For the COVID-19 response, we have experienced challenges around supply chains and making sure that our producers have the materials they need to make the vaccines.

As we think about manufacturing capacity for the future, we need to recognize the factors that have come together to produce the outcomes we don't like in the current response—the fact that a handful of countries and regions have dominated the vaccine supply—and take these into account as we develop solutions for the future. And whatever solutions we propose need to be sustainable. There will undoubtedly be a strong desire by many countries to have their own vaccine manufacturing facilities. But the question is, how many facilities can the world support and sustain? A facility that is built and then mothballed to wait until the next pandemic is not going to be very functional. So we have to balance across those dilemmas and find solutions that maximize our ability to scale up rapidly when necessary but that can also produce vaccines sustainably when we are not responding to a global crisis.

Also, as we think about global manufacturing capacity, we shouldn't do it with blinders on. We won't necessarily have to be able to vaccinate eight billion people every time a pandemic threat emerges. If we detect it early enough and use an empowered public-health enterprise to respond and suppress transmission—while we do the compressed-vaccine development and bring countermeasures in—maybe we only need 100 million doses. We shouldn't just focus on our productive capacity for vaccines without recognizing that we need to be able to address pandemics as efficiently as possible.

ChatGPT content

There are several challenges to increasing pandemic preparedness. One of the biggest challenges is the lack of political will or funding to prioritize pandemic preparedness efforts. Preparing for a pandemic can be expensive, and it is often difficult to justify the cost of such efforts until a pandemic is already underway.

Another challenge is the lack of coordination and cooperation among different levels of government, as well as between governments and other stakeholders such as international organizations, non-governmental organizations, and the private sector. Effective pandemic preparedness requires coordination across a wide range of sectors, and this can be difficult to achieve in practice.

A third challenge is the difficulty of predicting and preparing for future pandemics. There are many different factors that can contribute to the emergence and spread of a pandemic, and it is often difficult to anticipate which diseases will pose the greatest threat in the future. This makes it challenging to prioritize preparedness efforts and allocate resources effectively.

Finally, a lack of public understanding and engagement can also be a challenge to pandemic preparedness. Many people may not understand the importance of preparing for a pandemic or may not be aware of the steps they can take to protect themselves and others. This can make it difficult to implement effective prevention and control measures.

ChatGPT screenshot

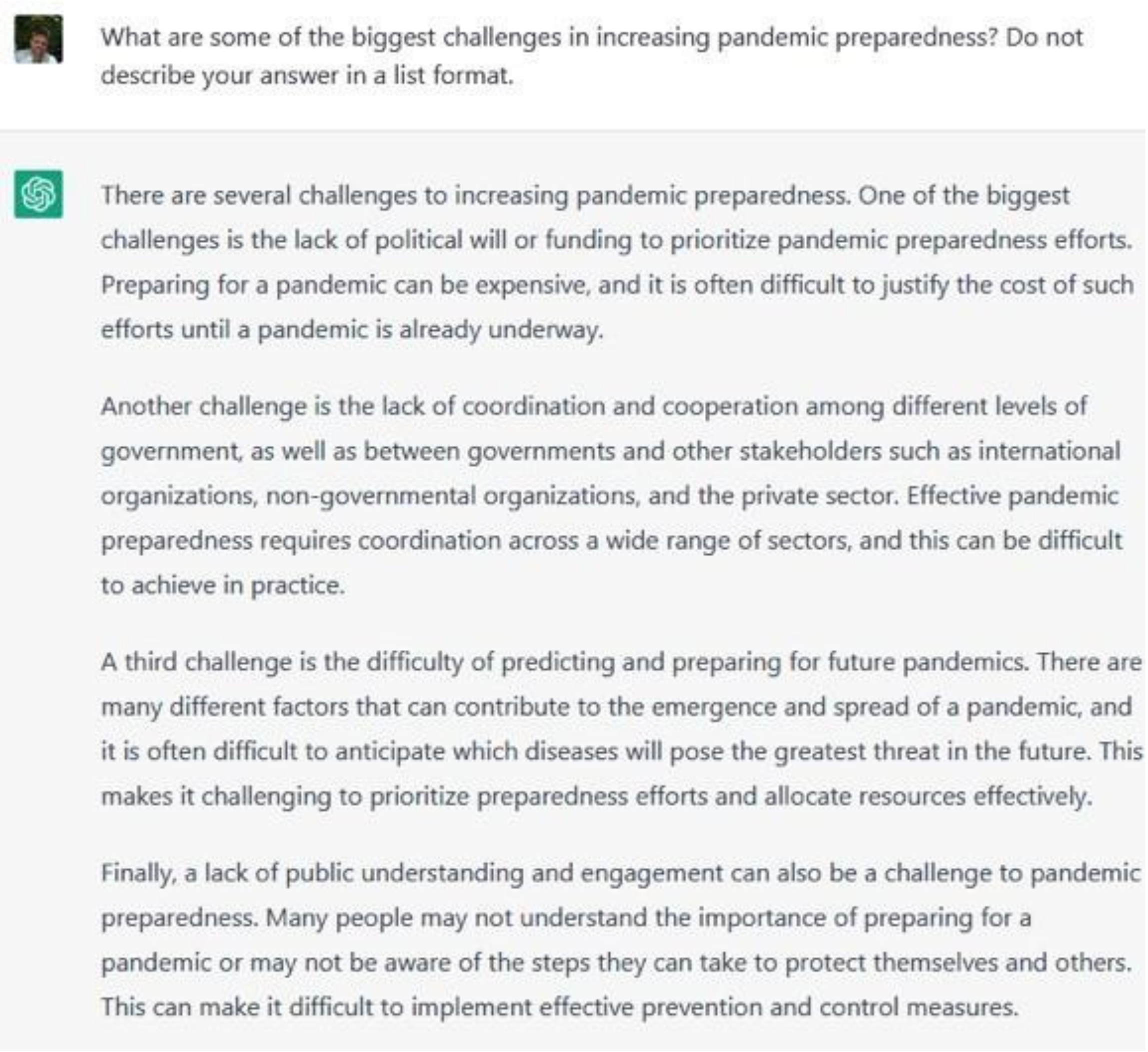

What are some of the biggest challenges in increasing pandemic preparedness? Do not describe your answer in a list format.

There are several challenges to increasing pandemic preparedness. One of the biggest challenges is the lack of political will or funding to prioritize pandemic preparedness efforts. Preparing for a pandemic can be expensive, and it is often difficult to justify the cost of such efforts until a pandemic is already underway.

Another challenge is the lack of coordination and cooperation among different levels of government, as well as between governments and other stakeholders such as international organizations, non-governmental organizations, and the private sector. Effective pandemic preparedness requires coordination across a wide range of sectors, and this can be difficult to achieve in practice.

A third challenge is the difficulty of predicting and preparing for future pandemics. There are many different factors that can contribute to the emergence and spread of a pandemic, and it is often difficult to anticipate which diseases will pose the greatest threat in the future. This makes it challenging to prioritize preparedness efforts and allocate resources effectively.

Finally, a lack of public understanding and engagement can also be a challenge to pandemic preparedness. Many people may not understand the importance of preparing for a pandemic or may not be aware of the steps they can take to protect themselves and others. This can make it difficult to implement effective prevention and control measures.

Please propose solutions that would mitigate the impacts of global warming, cut greenhouse gas emissions and help us reach our net zero goals.

From: Katharine Hayhoe: 'A thermometer is not liberal or conservative' | Climate science | The Guardian

Katharine Hayhoe (atmospheric scientist and director of the Climate Science Center at Texas Tech University. She has contributed to more than 125 scientific papers and won numerous prizes for her science communication work)

The most important thing is to accelerate the realisation that we have to act. This means connecting the dots to show that the impacts are not distant any more: they are here and they affect our lives. It also means talking about solutions. The technology and knowledge are there. The economics already make sense. In Texas, where I live, the biggest military base, Fort Hood, switched last year to renewables because they were cheaper than natural gas. And finally, it means weaning ourselves off fossil fuels, which is challenged by the fact that the majority of the world's richest companies have made their money from the fossil fuel economy – so the majority of the wealth and power remains in their hands.

ChatGPT content

There are several measures that can be taken to mitigate the impacts of global warming, cut greenhouse gas emissions, and help us reach our net zero goals.

One approach is to transition to clean, renewable energy sources such as solar, wind, and hydroelectric power. This can be achieved through a variety of means, including increasing the use of renewable energy in electricity generation, implementing policies to support the development and deployment of clean energy technologies, and providing financial incentives for individuals and businesses to adopt clean energy practices.

Another approach is to improve energy efficiency in buildings, transportation, and industrial processes. This can be done through the use of energy-efficient appliances, vehicles, and industrial equipment, as well as through the implementation of energy-saving policies and practices.

Conserving and protecting forests and other natural habitats is also important, as these ecosystems absorb and store carbon dioxide from the atmosphere. This can be done through

reforestation and afforestation projects, as well as through conservation efforts to protect existing forests and other natural habitats.

In addition, reducing methane emissions from agriculture, waste, and other sources can have a significant impact on global warming. Methane is a potent greenhouse gas, and reducing its emissions can help to slow the rate of global warming.

Finally, it is important to engage in international cooperation and support the development of global climate policies and agreements. Working together with other countries can help to amplify the impact of individual efforts and ensure that progress is made towards our net zero goals.

ChatGPT screenshot

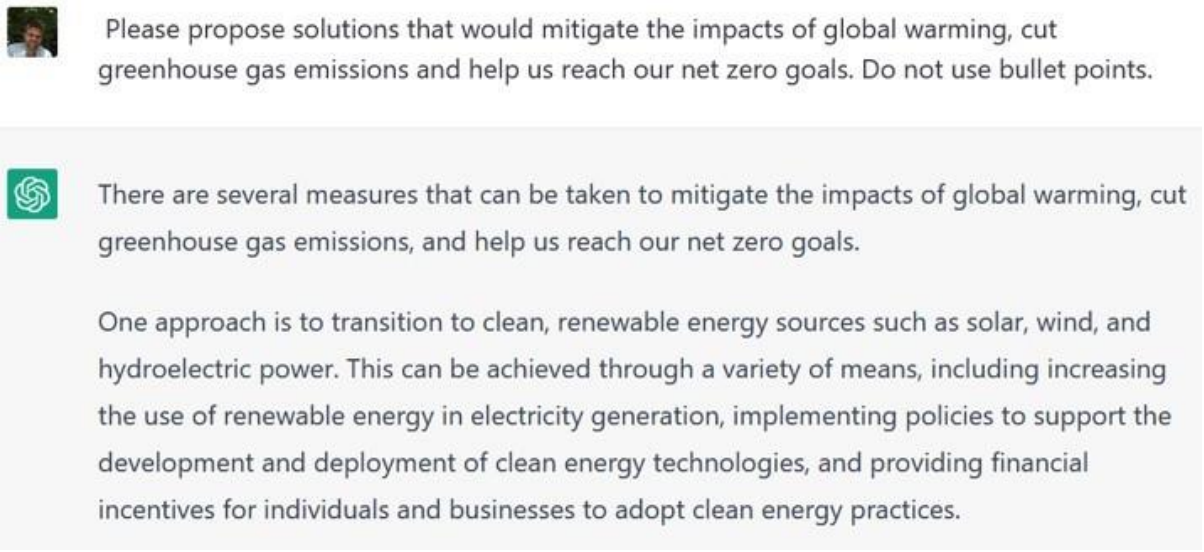

Please propose solutions that would mitigate the impacts of global warming, cut greenhouse gas emissions and help us reach our net zero goals. Do not use bullet points.

There are several measures that can be taken to mitigate the impacts of global warming, cut greenhouse gas emissions, and help us reach our net zero goals.

One approach is to transition to clean, renewable energy sources such as solar, wind, and hydroelectric power. This can be achieved through a variety of means, including increasing the use of renewable energy in electricity generation, implementing policies to support the development and deployment of clean energy technologies, and providing financial incentives for individuals and businesses to adopt clean energy practices.

Another approach is to improve energy efficiency in buildings, transportation, and industrial processes. This can be done through the use of energy-efficient appliances, vehicles, and industrial equipment, as well as through the implementation of energy-saving policies and practices.

Conserving and protecting forests and other natural habitats is also important, as these ecosystems absorb and store carbon dioxide from the atmosphere. This can be done through reforestation and afforestation projects, as well as through conservation efforts to protect existing forests and other natural habitats.

In addition, reducing methane emissions from agriculture, waste, and other sources can have a significant impact on global warming. Methane is a potent greenhouse gas, and reducing its emissions can help to slow the rate of global warming.

Finally, it is important to engage in international cooperation and support the development of global climate policies and agreements. Working together with other countries can help to amplify the impact of individual efforts and ensure that progress is made towards our net zero goals.

What do you see as the overall impact AI will have on the workforce, and how can we prepare for these changes?

From: Five Questions for Erik Brynjolfsson

Erik Brynjolfsson (Professor and Senior Fellow at the Stanford Institute for Human-Centered AI (HAI), and Director of the Stanford Digital Economy Lab)

As artificial intelligence becomes more powerful, it will inevitably lead to profound changes in our economy and society. Some of these are already beginning, as AI affects an increasing number of tasks and jobs. Over the next 10 years, more kinds of work will be transformed. This will reduce demand for many different skills, like the people working in call centers and driving trucks, and increase demand for other kinds of skills like creative work and interpersonal and caring professions. I see this as the greatest challenge for our society over the next decade.

In the longer term, we will face other types of challenges, but for now I think we should focus on finding ways that we can use technology to create widely shared prosperity that benefits the many and not just a few. This involves rethinking our educational system; boosting entrepreneurship, so we can invent more new products, services, and jobs that will employ people; making the tax system less skewed towards the wealthy; and making new investments in research and development and infrastructure. Ultimately, I think these technologies can be the best things that happened to our society, if we make the right complementary investments and rethink our organizations and institutions.

ChatGPT content

Artificial intelligence (AI) is likely to have a significant impact on the workforce, as it has the potential to automate many tasks that are currently performed by humans. This could lead to job displacement in certain industries, particularly in roles that involve repetitive or predictable tasks. However, it is important to note that AI is also expected to create new job opportunities, particularly in fields such as data science and AI development.

To prepare for these changes, it is important for individuals to continuously update their skills and consider pursuing education and training in areas that are likely to be in demand in the future. It is also important for organizations to consider how they can support their employees in developing new skills and transitioning to new roles as necessary. In addition, there is a need for policies and programs that can help individuals who may be impacted by job displacement due to AI to find new employment or retrain for new roles.

ChatGPT screenshot

What do you see as the overall impact AI will have on the workforce, and how can we prepare for these changes? Provide answer shorter than 200 words.\

Artificial intelligence (AI) is likely to have a significant impact on the workforce, as it has the potential to automate many tasks that are currently performed by humans. This could lead to job displacement in certain industries, particularly in roles that involve repetitive or predictable tasks. However, it is important to note that AI is also expected to create new job opportunities, particularly in fields such as data science and AI development.

To prepare for these changes, it is important for individuals to continuously update their skills and consider pursuing education and training in areas that are likely to be in demand in the future. It is also important for organizations to consider how they can support their employees in developing new skills and transitioning to new roles as necessary. In addition, there is a need for policies and programs that can help individuals who may be impacted by job displacement due to AI to find new employment or retrain for new roles.

Studies 2a and 2b

How can I start exercising more regularly?

ChatGPT content

Starting an exercise routine can be challenging, but it can also be very rewarding. Here are a few tips to help you get started:

1. Set specific, achievable goals. Instead of saying, "I want to exercise more," decide on a specific goal, such as, "I want to walk for 30 minutes every weekday morning."
2. Make a plan. Decide when, where, and how you will exercise. Will you walk or run? Will you go to the gym or exercise at home? Will you exercise in the morning or in the evening? Write down your plan and stick to it as best as you can.
3. Start small. If you're new to exercise, or if you haven't exercised in a while, it's important to start slowly and gradually increase the intensity and duration of your workouts. Start with 5-10 minutes of exercise per day and work your way up.
4. Find activities you enjoy. The more you enjoy an activity, the more likely you are to stick with it. Experiment with different types of exercise to find what you like: cycling, swimming, dancing, hiking, etc.

5. Exercise with a friend. Find a friend or family member to exercise with you. They can provide motivation and accountability.
6. Track your progress. Keep track of your exercise routine and your progress toward your goal. This will help you stay motivated and see the progress you're making.
7. Be consistent and patient. It may take time to build the habit of regular exercise, so be patient with yourself. Also, Try not to miss more than 2 days of exercise in a row, and make sure to find something active to do on days when you’re not able to do your regular routine.

Remember that consistency is key when trying to establish an exercise routine. It can take time and effort to make exercise a regular part of your life, but the benefits, both for your physical and mental health, are well worth it.

ChatGPT screenshot

How can I start exercising more regularly?

Starting an exercise routine can be challenging, but it can also be very rewarding. Here are a few tips to help you get started:

1. Set specific, achievable goals. Instead of saying, "I want to exercise more," decide on a specific goal, such as, "I want to walk for 30 minutes every weekday morning."
2. Make a plan. Decide when, where, and how you will exercise. Will you walk or run? Will you go to the gym or exercise at home? Will you exercise in the morning or in the evening? Write down your plan and stick to it as best as you can.
3. Start small. If you're new to exercise, or if you haven't exercised in a while, it's important to start slowly and gradually increase the intensity and duration of your workouts. Start with 5-10 minutes of exercise per day and work your way up.
4. Find activities you enjoy. The more you enjoy an activity, the more likely you are to stick with it. Experiment with different types of exercise to find what you like: cycling, swimming, dancing, hiking, etc.
5. Exercise with a friend. Find a friend or family member to exercise with you. They can provide motivation and accountability.
6. Track your progress. Keep track of your exercise routine and your progress toward your goal. This will help you stay motivated and see the progress you're making.
7. Be consistent and patient. It may take time to build the habit of regular exercise, so be patient with yourself. Also, Try not to miss more than 2 days of exercise in a row, and make sure to find something active to do on days when you're not able to do your regular routine.

Remember that consistency is key when trying to establish an exercise routine. It can take time and effort to make exercise a regular part of your life, but the benefits, both for your physical and mental health, are well worth it.

How can I manage to quit smoking?

ChatGPT content

Quitting smoking is a difficult process, but it is possible with the right mindset and tools. Here are a few tips that may help you:

1. Make a plan: Decide on a quit date and stick to it. Choose a method of quitting that works for you, such as nicotine replacement therapy or prescription medication.
2. Get support: Having a support system in place can make a big difference in your success. Consider enlisting the help of a friend, family member, or support group.
3. Find healthy ways to cope with cravings: When a craving strikes, try to find a healthy way to cope with it. Go for a walk, drink a glass of water, or try deep breathing exercises.
4. Don't be too hard on yourself: Quitting smoking is a challenging process and it's normal to have setbacks. If you slip up, don't be too hard on yourself. Just get back on track and keep trying.
5. Stay positive: Remember that quitting smoking is one of the best things you can do for your health. It may be difficult at times, but the benefits are worth it.

If you need additional support, consider talking to a healthcare provider or a smoking cessation program. They can provide you with the guidance and resources you need to successfully quit smoking.

ChatGPT screenshot

How can I manage to quit smoking?

Quitting smoking is a difficult process, but it is possible with the right mindset and tools. Here are a few tips that may help you:

1. Make a plan: Decide on a quit date and stick to it. Choose a method of quitting that works for you, such as nicotine replacement therapy or prescription medication.
2. Get support: Having a support system in place can make a big difference in your success. Consider enlisting the help of a friend, family member, or support group.
3. Find healthy ways to cope with cravings: When a craving strikes, try to find a healthy way to cope with it. Go for a walk, drink a glass of water, or try deep breathing exercises.
4. Don't be too hard on yourself: Quitting smoking is a challenging process and it's normal to have setbacks. If you slip up, don't be too hard on yourself. Just get back on track and keep trying.
5. Stay positive: Remember that quitting smoking is one of the best things you can do for your health. It may be difficult at times, but the benefits are worth it.

If you need additional support, consider talking to a healthcare provider or a smoking cessation program. They can provide you with the guidance and resources you need to successfully quit smoking.

How can I start eating healthier?

ChatGPT content

1. Start by setting realistic goals for yourself. This could be something as simple as incorporating more fruits and vegetables into your diet.

2. Make a grocery list and stick to it. Avoid impulse buys and only purchase items that align with your healthy eating goals.

3. Plan your meals in advance and make sure to include a variety of healthy options. This can help prevent the temptation to eat fast food or order takeout.

4. Incorporate more plant-based options into your diet. This can include beans, nuts, and whole grains.

5. Keep healthy snacks on hand, such as fruits, vegetables, and nuts, to avoid snacking on processed foods.

6. Try to cook at home as often as possible. This allows you to control the ingredients and avoid processed foods.

7. Drink plenty of water and limit your intake of sugary drinks.

8. Finally, don't be too hard on yourself. Making changes to your diet takes time, and it's important to be patient and kind to yourself throughout the process.

ChatGPT screenshot

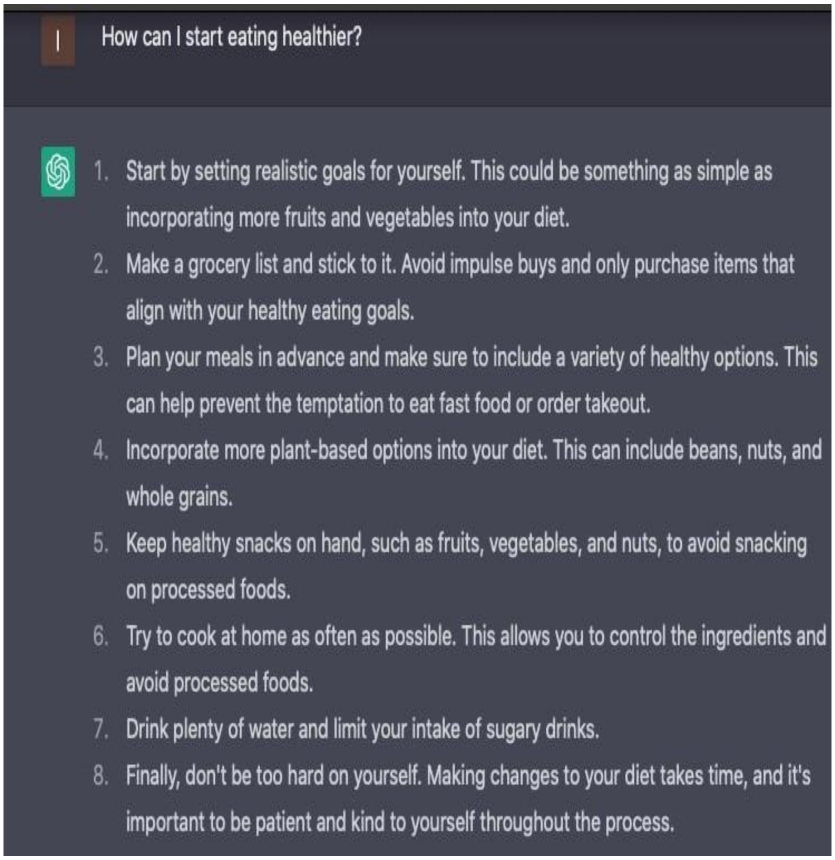

I How can I start eating healthier?

1. Start by setting realistic goals for yourself. This could be something as simple as incorporating more fruits and vegetables into your diet.
2. Make a grocery list and stick to it. Avoid impulse buys and only purchase items that align with your healthy eating goals.
3. Plan your meals in advance and make sure to include a variety of healthy options. This can help prevent the temptation to eat fast food or order takeout.
4. Incorporate more plant-based options into your diet. This can include beans, nuts, and whole grains.
5. Keep healthy snacks on hand, such as fruits, vegetables, and nuts, to avoid snacking on processed foods.
6. Try to cook at home as often as possible. This allows you to control the ingredients and avoid processed foods.
7. Drink plenty of water and limit your intake of sugary drinks.
8. Finally, don't be too hard on yourself. Making changes to your diet takes time, and it's important to be patient and kind to yourself throughout the process.

How can I reduce the time I spend on my smartphone?

ChatGPT content

1. Set specific times of the day to check your phone: Choose a few specific times of the day to check your phone, such as when you wake up, before bed, and during lunchtime.

2. Use apps to track your usage: There are various apps available that track your phone usage, such as Moment, QualityTime, and BreakFree. These apps can help you monitor your habits and make changes to reduce your usage.

3. Turn off notifications: Disabling notifications for apps that aren't essential will reduce the amount of time you spend checking your phone.

4. Find alternative activities: Instead of checking your phone, find other activities to do such as reading a book, exercising, or spending time with friends.

5. Use the "Do Not Disturb" feature: This feature is available on most smartphones and blocks notifications and calls when enabled. Use this feature when you need to focus on work or other important tasks.

6. Keep your phone out of reach: Keep your phone out of reach when you don't need it, such as in another room or in your bag, so you're less likely to check it frequently.

ChatGPT screenshot

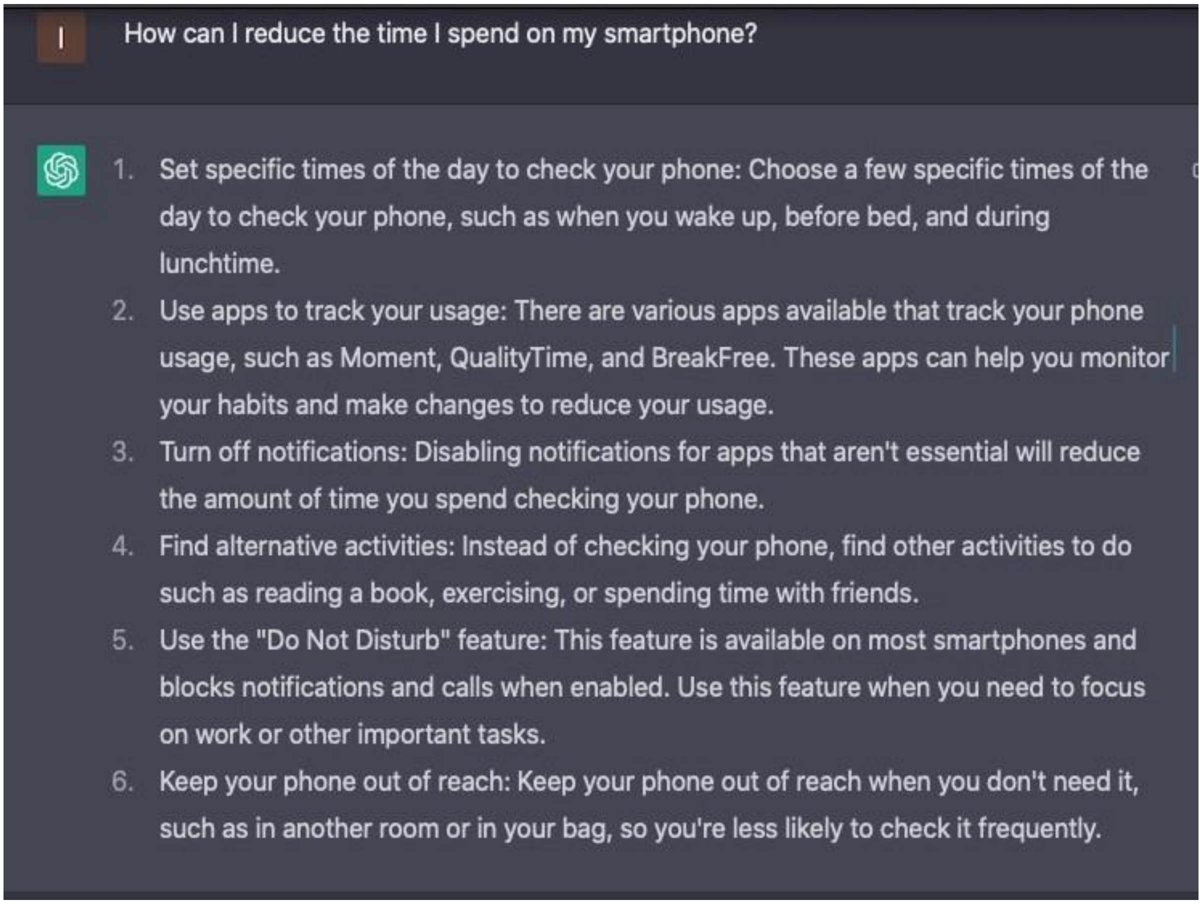

How can I save more money?

ChatGPT content

1. Create a budget and stick to it: Set a budget for your monthly expenses and try to stick to it as much as possible. This will help you keep track of where your money is going and make adjustments as needed.

2. Cut back on unnecessary expenses: Review your expenses and see where you can cut back. For example, you can cancel subscriptions or memberships you don't use, or find cheaper alternatives for things like entertainment or transportation.

3. Save a portion of your income: Set aside a certain percentage of your income for savings each month. You can also automate your savings by setting up automatic transfers from your checking account to your savings account.

4. Find ways to earn extra income: Look for opportunities to earn extra money through side hustles, such as freelancing, renting out a spare room, or selling items you no longer need.

5. Invest wisely: Consider investing in stocks, mutual funds, or other forms of investment that can help grow your savings over time. However, make sure to do your research before investing and only invest money you can afford to lose.

6. Seek professional advice: If you need help managing your finances, consider seeking advice from a financial advisor or a credit counselor. They can help you create a plan to achieve your financial goals and provide guidance on how to save more money.

ChatGPT screenshot

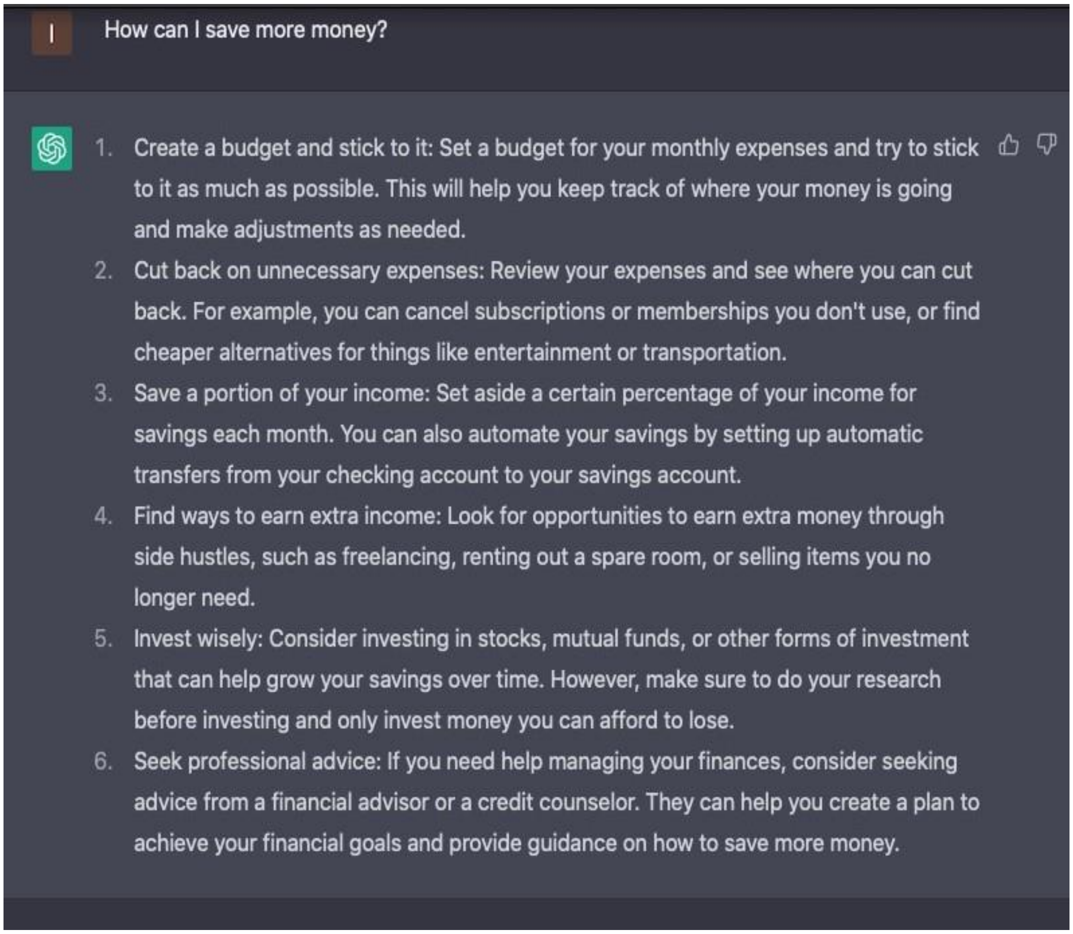
How can I save more money?

1. Create a budget and stick to it: Set a budget for your monthly expenses and try to stick to it as much as possible. This will help you keep track of where your money is going and make adjustments as needed.
2. Cut back on unnecessary expenses: Review your expenses and see where you can cut back. For example, you can cancel subscriptions or memberships you don't use, or find cheaper alternatives for things like entertainment or transportation.
3. Save a portion of your income: Set aside a certain percentage of your income for savings each month. You can also automate your savings by setting up automatic transfers from your checking account to your savings account.
4. Find ways to earn extra income: Look for opportunities to earn extra money through side hustles, such as freelancing, renting out a spare room, or selling items you no longer need.
5. Invest wisely: Consider investing in stocks, mutual funds, or other forms of investment that can help grow your savings over time. However, make sure to do your research before investing and only invest money you can afford to lose.
6. Seek professional advice: If you need help managing your finances, consider seeking advice from a financial advisor or a credit counselor. They can help you create a plan to achieve your financial goals and provide guidance on how to save more money.

How can I have a positive impact on the world?

ChatGPT content

1. Volunteer: You can make a positive impact by volunteering your time and skills to organizations that support causes you believe in.

2. Support sustainable practices: Supporting sustainable practices and products can help reduce the impact of human activities on the environment.

3. Donate to charity: Giving to charity can help support various causes, from fighting poverty to protecting the environment.

4. Be an advocate: You can raise awareness about important issues by being an advocate for causes you believe in.

5. Spread kindness: Small acts of kindness can have a ripple effect and can have a positive impact on the world.

6. Support small businesses: Supporting small businesses can help boost the local economy and create jobs.

7. Educate yourself: Learning about different cultures, issues, and perspectives can help you to better understand the world and how to make a positive impact.

8. Live a sustainable lifestyle: Eating healthy, conserving energy and water, recycling, and reducing waste can help reduce the impact of human activities on the environment.

9. Be a responsible consumer: Be mindful of the products you buy, and look for companies that have ethical and sustainable practices.

10. Be politically active: You can make a positive impact by staying informed about issues and participating in the political process.

ChatGPT screenshot

I How can I have a positive impact on the world?

1. Volunteer: You can make a positive impact by volunteering your time and skills to organizations that support causes you believe in.
2. Support sustainable practices: Supporting sustainable practices and products can help reduce the impact of human activities on the environment.
3. Donate to charity: Giving to charity can help support various causes, from fighting poverty to protecting the environment.
4. Be an advocate: You can raise awareness about important issues by being an advocate for causes you believe in.
5. Spread kindness: Small acts of kindness can have a ripple effect and can have a positive impact on the world.
6. Support small businesses: Supporting small businesses can help boost the local economy and create jobs.
7. Educate yourself: Learning about different cultures, issues, and perspectives can help you to better understand the world and how to make a positive impact.
8. Live a sustainable lifestyle: Eating healthy, conserving energy and water, recycling, and reducing waste can help reduce the impact of human activities on the environment.
9. Be a responsible consumer: Be mindful of the products you buy, and look for companies that have ethical and sustainable practices.
10. Be politically active: You can make a positive impact by staying informed about issues and participating in the political process.

Supplementary Tables and Figures

For the exact code to re-create all analyses, tables and figures please refer to: https://osf.io/6ptx8

Study 1

Supplementary Table 1

Results of author evaluation controlled for demographic variables in Study 1

	Df	Sum Sq	Mean Sq	F value	Pr(>F)	p Bonferroni Correction
Author Identity	1	14.764	14.764	12.653	0.000	.004*
Transparency of the Author	1	3.780	3.780	3.240	0.072	.722
Topic of the Content	4	1.451	0.363	0.311	0.871	1.000
Gender	3	6.320	2.107	1.805	0.144	1.000
Age	1	0.122	0.122	0.104	0.747	1.000
Education	1	3.915	3.915	3.355	0.067	.673
Author Identity*Transparency of the Author	1	17.206	17.206	14.746	0.000	.001***
Author Identity* Topic of the Content	4	2.781	0.695	0.596	0.666	1.000
Transparency of the Author*Topic of the Content	4	3.775	0.944	0.809	0.520	1.000
Author Identity*Transparency of the Author* Topic of the Content	4	2.902	0.726	0.622	0.647	1.000
Residuals	976	1138.838	1.167	NA	NA	NA

Note. **p* < .05, ***p* < .01, ****p* < .001, Df = Degrees of freedom, Sum sq = Sum of squares, Mean Sq = Mean Sum of Squares.

Supplementary Table 2

Effect of subjective knowledge on who is assumed to be author in Study 1

	Df	Sum Sq	Mean Sq	F value	Pr(>F)	p Bonferroni Correction
Author Identity	1	0.542	0.542	0.242	0.624	1.000
Subjective Knowledge	1	2.258	2.258	1.008	0.318	.953
Author Identity* Subjective Knowledge	1	0.134	0.134	0.060	0.807	1.000
Residuals	112	250.894	2.240	NA	NA	NA

Note. Df = Degrees of freedom, Sum sq = Sum of squares, Mean Sq = Mean Sum of Squares.

Supplementary Figure 1

Content evaluation across contexts by author transparency in Study 1 (N = 1003).

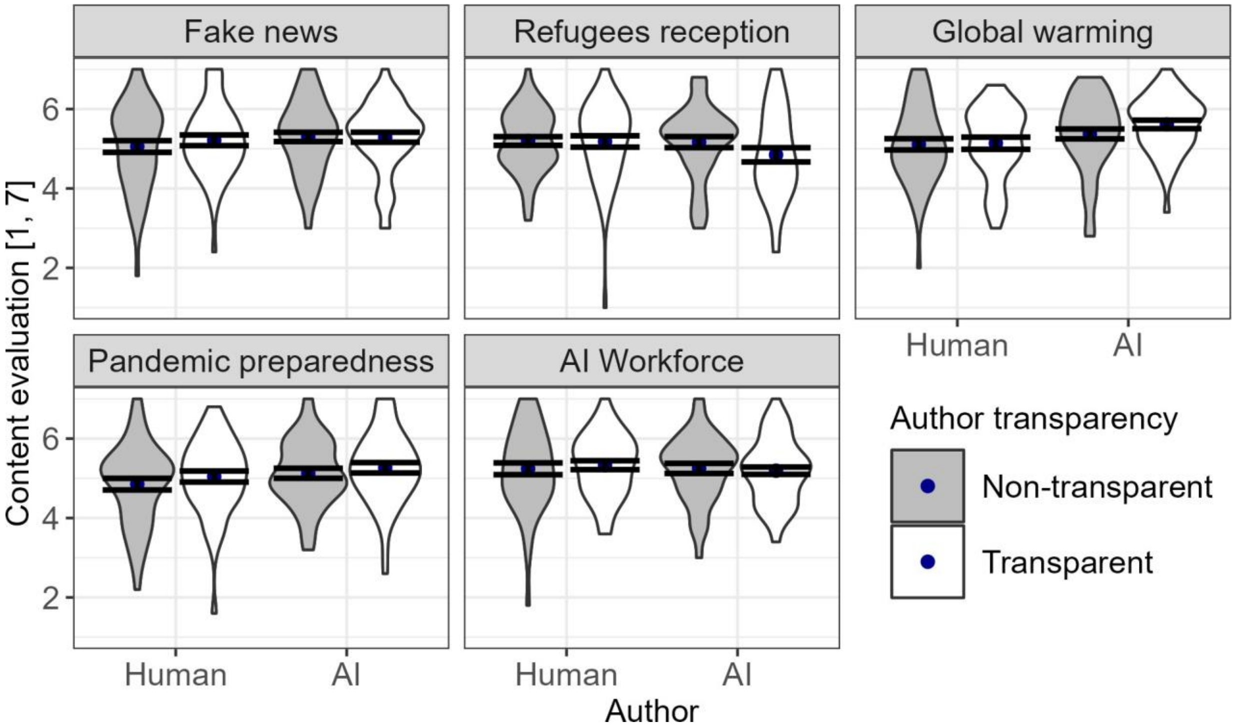

Note. Content evaluation was calculated as the mean value from five response items (see Methods section of the main text). Dots indicate mean values, error bars represent 95% confidence intervals, and gray/white areas display kernel densities.

Supplementary Figure 2

Intention to share the content with others across contexts by author transparency in Study 1 (*N* = 1003).

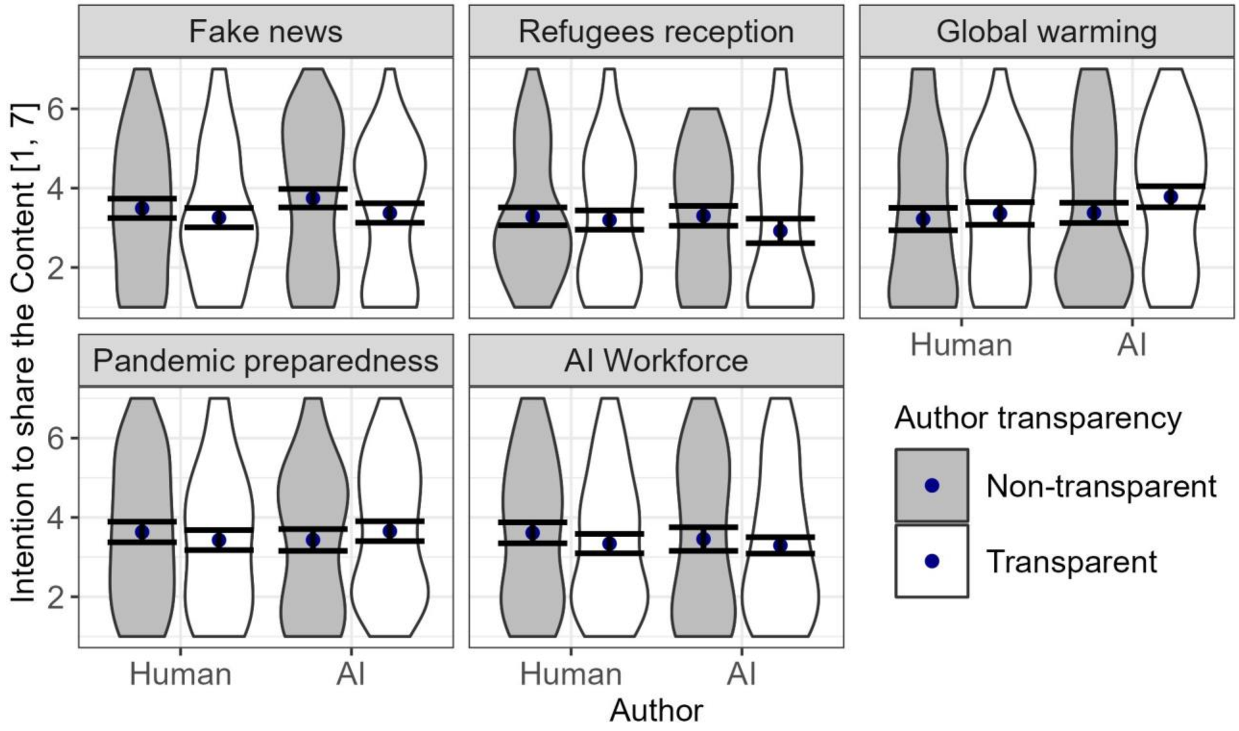

Note. The intention was calculated as the mean value from one response item (see Methods section of the main text). Dots indicate mean values, error bars represent 95% confidence intervals, and gray/white areas display kernel densities.

Supplementary Figure 3

Distribution of responses for the outcome variable Author competence in Study 1 (N = 1003).

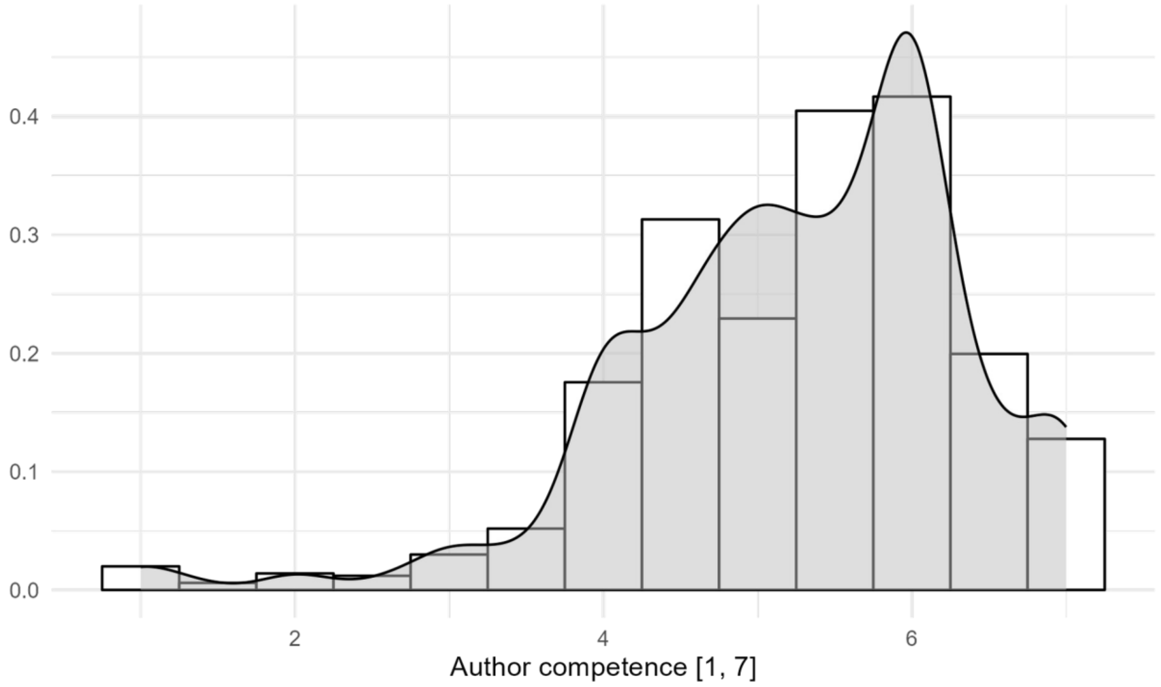

Supplementary Figure 4

Distribution of responses for the outcome variable Content evaluation in Study 1 (*N* = 1003).

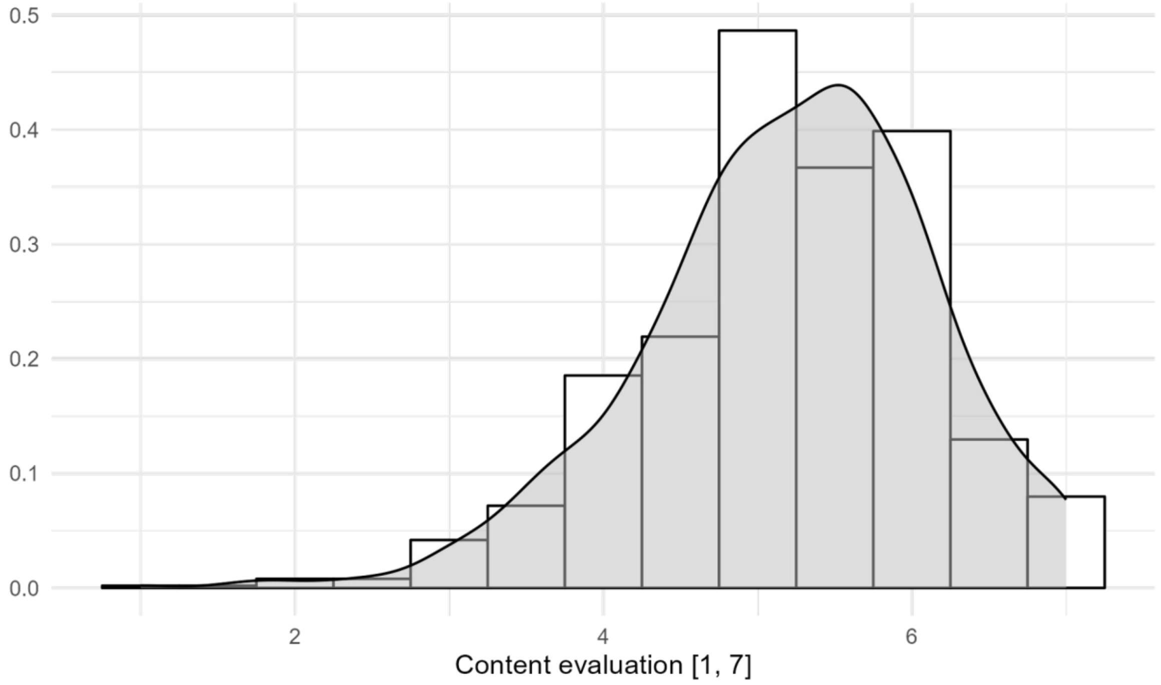

Supplementary Figure 5

Distribution of responses for the outcome variable Intention to share the content in Study 1 (N = 1003).

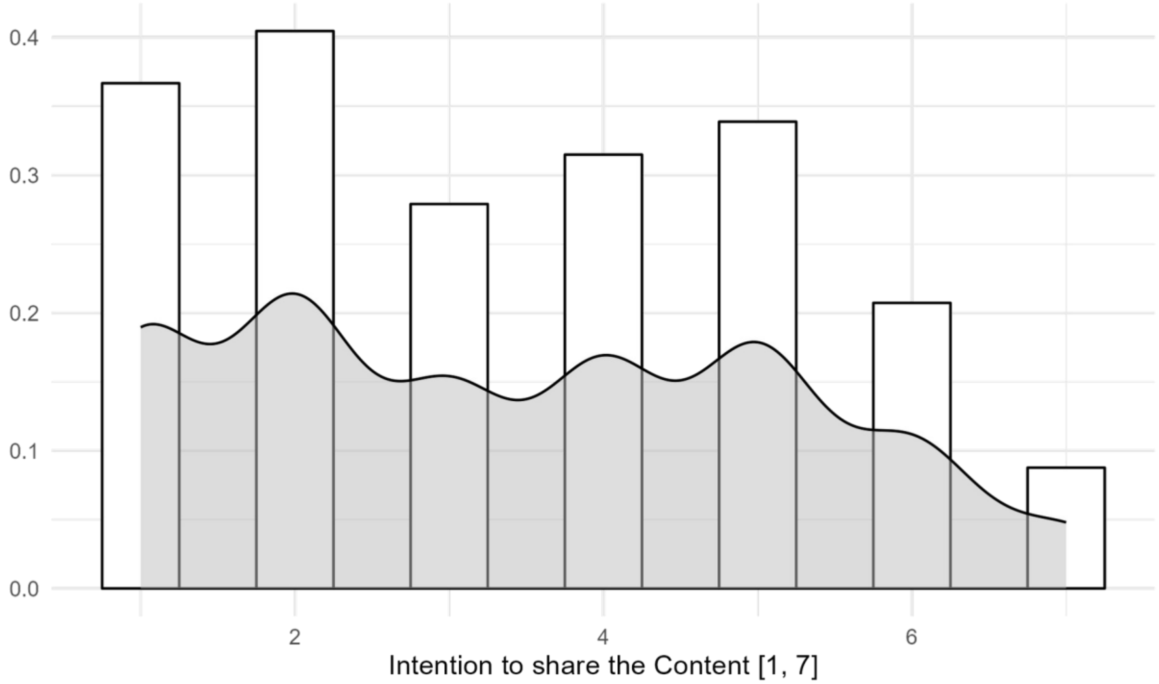

Supplementary Figure 6

Distribution of responses for the outcome variable Guess who is the author in Study 1 (N = 508).

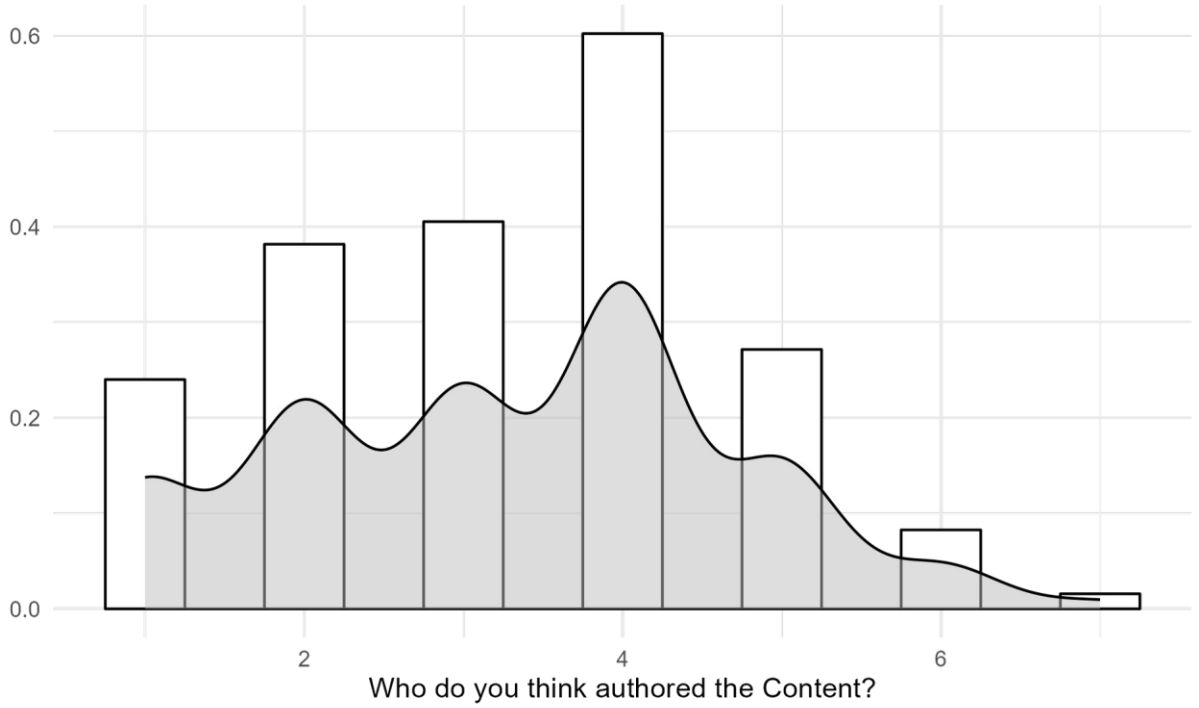

Note. Likert-scale ranging from 1 – *Definitely a human* to 7- *Definitely a artificial intelligence (AI)*.

Studies 2a and 2b

Supplementary Table 3

Results of Author evaluation controlled for demographic variables in Study 2a

	Df	Sum Sq	Mean Sq	F value	Pr(>F)
Author Transparency	1	23.529	23.529	19.965	.001***
Topic of Content	5	20.476	4.095	3.475	.004**
Age	1	1.601	1.601	1.359	.244
Gender	3	0.999	0.333	0.283	.838
Education	1	1.566	1.566	1.329	.250
Transparency*Topic	5	11.039	2.208	1.873	.097
Residuals	484	570.415	1.179	NA	NA

Note. *$p < .05$, **$p < .01$, ***$p < .001$, Df = Degrees of freedom. Sum sq = Sum of squares. Mean Sq = Mean Sum of Squares.

Supplementary Table 4

Results of text evaluation controlled for demographic variables in Study 2b

	Df	Sum Sq	Mean Sq	F value	Pr(>F)
Author Transparency	1	0.704	0.704	1.218	.270
Topic of Content	5	3.643	0.729	1.261	.279
Age	1	8.254	8.254	14.284	.001***
Gender	3	4.008	1.336	2.312	.075
Education	1	0.051	0.051	0.088	.767
Transparency*Topic	5	3.218	0.644	1.114	.351
Residuals	782	451.874	0.578	NA	NA

Note. *$p < .05$, **$p < .01$, ***$p < .001$, Df = Degrees of freedom. Sum sq = Sum of squares. Mean Sq = Mean Sum of Squares.

Supplementary Table 5

Results of author evaluation controlled for demographic variables in Study 2b

	Df	Sum Sq	Mean Sq	F value	Pr(>F)
Author Transparency	1	18.177	18.177	16.432	.001***
Topic of Content	5	16.615	3.323	3.004	.011*
Age	1	0.062	0.062	0.056	.813
Gender	3	15.711	5.237	4.734	.003**
Education	1	0.036	0.036	0.032	.858
Transparency*Topic	5	1.683	0.337	0.304	.910
Residuals	782	865.069	1.106	NA	NA

Note. *p < .05, **p < .01, ***p < .001, Df = Degrees of freedom. Sum sq = Sum of squares. Mean Sq = Mean Sum of Squares.

Supplementary Table 6

Results of behavioral intention controlled for demographic variables in Study 2b

	Df	Sum Sq	Mean Sq	F value	Pr(>F)
Author Transparency	1	4.459	4.459	2.615	.106
Topic of Content	5	77.669	15.534	9.111	.001***
Age	1	0.055	0.055	0.032	.857
Gender	3	24.116	8.039	4.715	.003**
Education	1	0.297	0.297	0.174	.677
Transparency*Topic	5	12.744	2.549	1.495	.189
Residuals	782	1333.336	1.705	NA	NA

Note. *p < .05, **p < .01, ***p < .001, Df = Degrees of freedom, Sum sq = Sum of squares, Mean Sq = Mean Sum of Squares.

Supplementary Table 7

Results of sharing intention controlled for demographic variables in Study 2b

	Df	Sum Sq	Mean Sq	F value	Pr(>F)
Author Transparency	1	3.326	3.326	0.853	.356
Topic of Content	5	18.748	3.750	0.962	.440
Age	1	5.263	5.263	1.350	.246
Gender	3	17.515	5.838	1.498	.214
Education	1	0.069	0.069	0.018	.894
Transparency*Topic	5	3.033	0.607	0.156	.978
Residuals	782	3047.843	3.897	NA	NA

Note. Df = Degrees of freedom, Sum sq = Sum of squares, Mean Sq = Mean Sum of Squares.

Supplementary Figure 7

Content evaluation across contexts by author transparency in Studies 2a (panel a, *N* = 501) and 2b (panel b, *N* = 800).

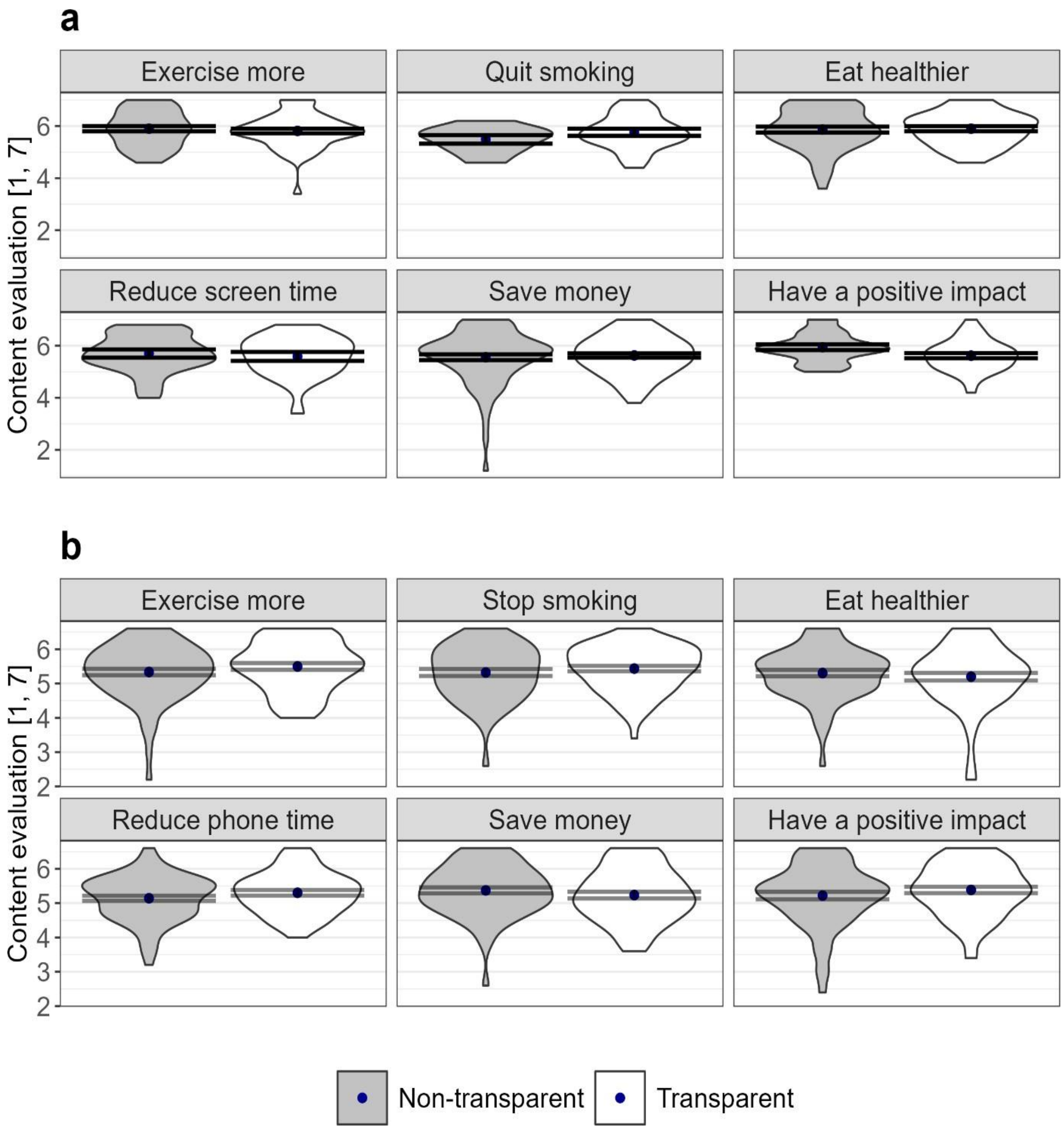

Note. Content evaluation was calculated as the mean value from 5 response items (see Method section of the main text). Dots indicate mean values, error bars represent 95% confidence intervals, and gray/white areas display kernel densities.

Supplementary Figure 8

Intention to follow the advice given across contexts by author transparency in Studies 2a (panel a, N = 501) and 2b (panel b, N = 800).

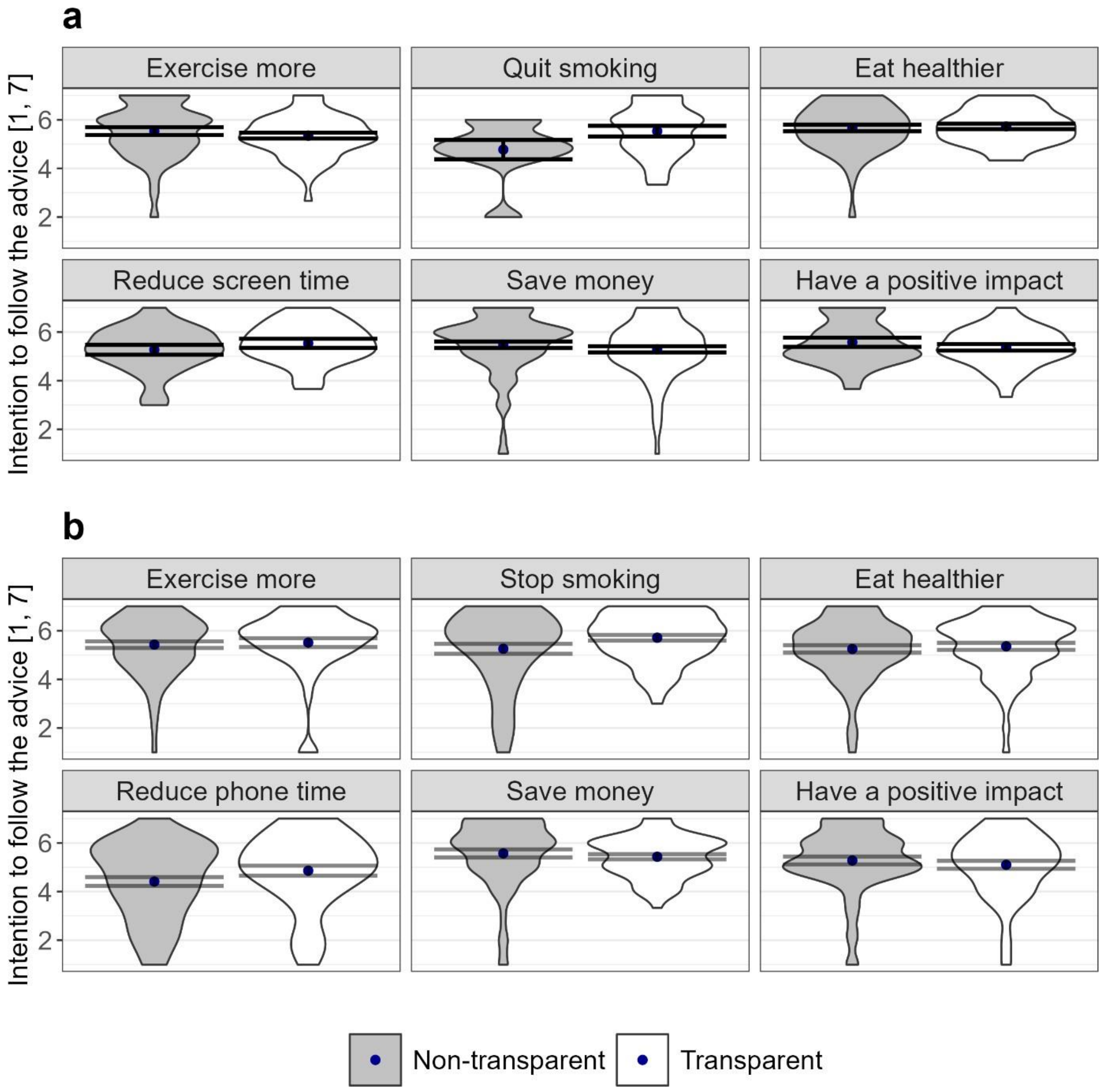

Note. Intention to follow the advice was calculated as the mean value from 3 response items (see Method section of the main text). Dots indicate mean values, error bars represent 95% confidence intervals, and gray/white areas display kernel densities.

Supplementary Figure 9

Intention to share the content given across contexts by author transparency in Studies 2a (panel a, N = 501) and 2b (panel b, N = 800).

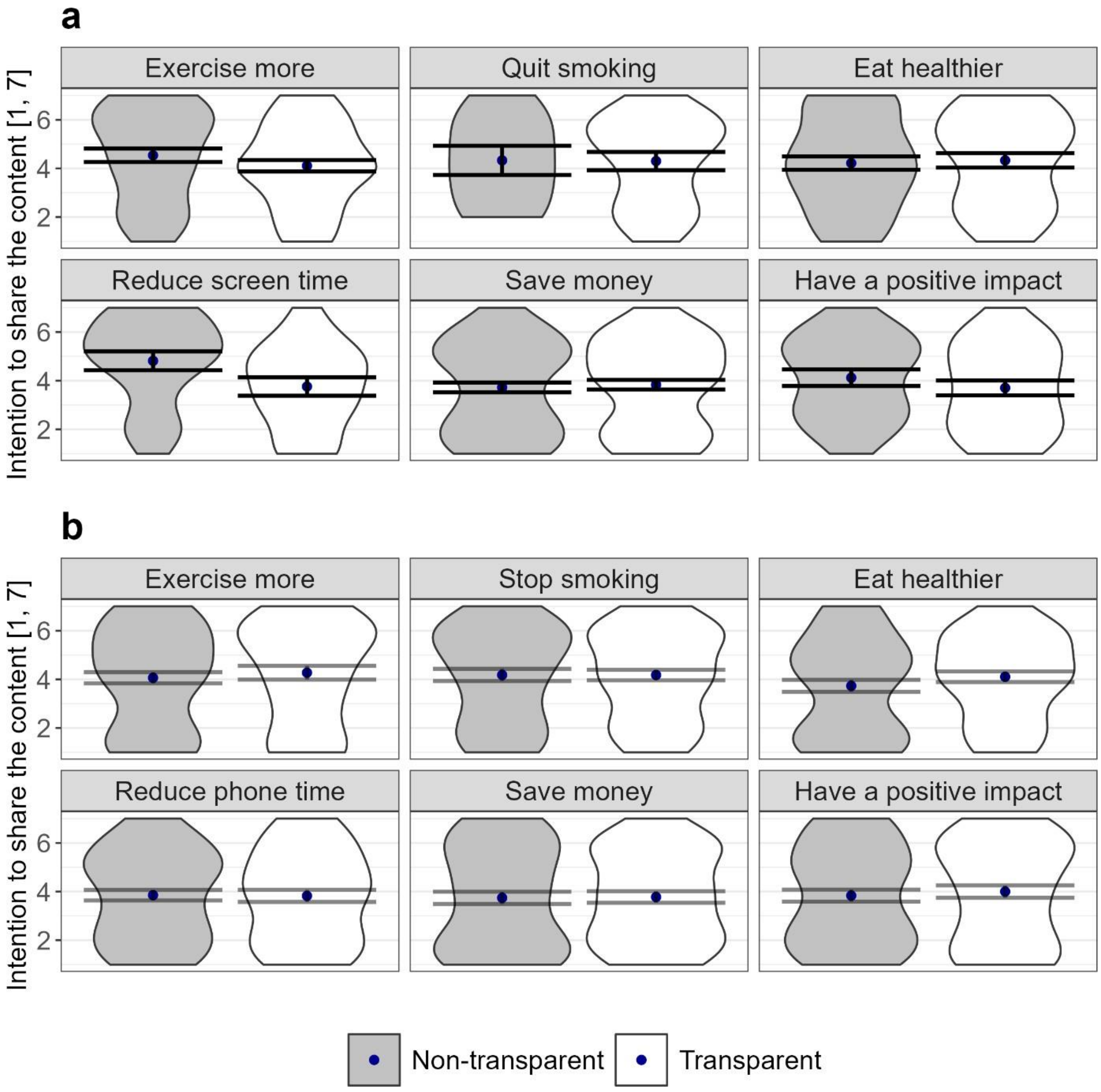

Note. Dots indicate mean values, error bars represent 95% confidence intervals, and gray/white areas display kernel densities.

Supplementary Figure 10

Distribution of responses for the outcome variable Author competence in Study 2a (N = 501).

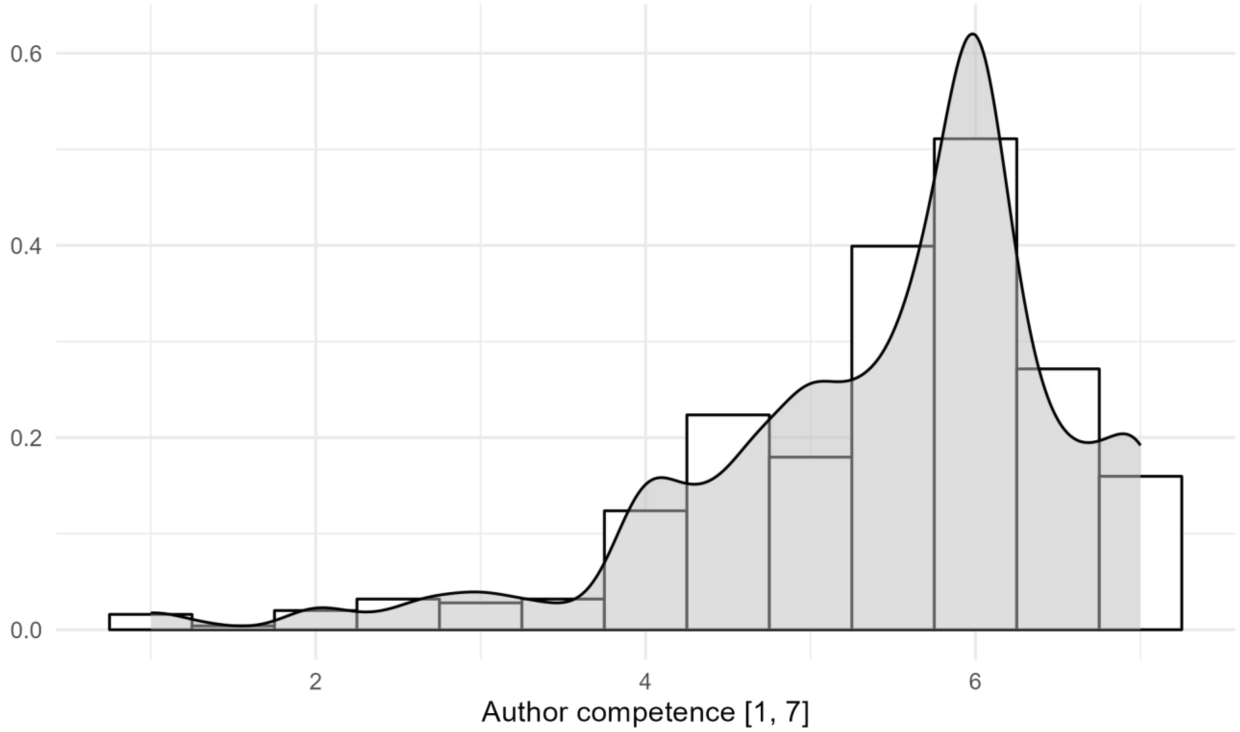

Supplementary Figure 11

Distribution of responses for the outcome variable Content evaluation in Study 2a (*N* = 501).

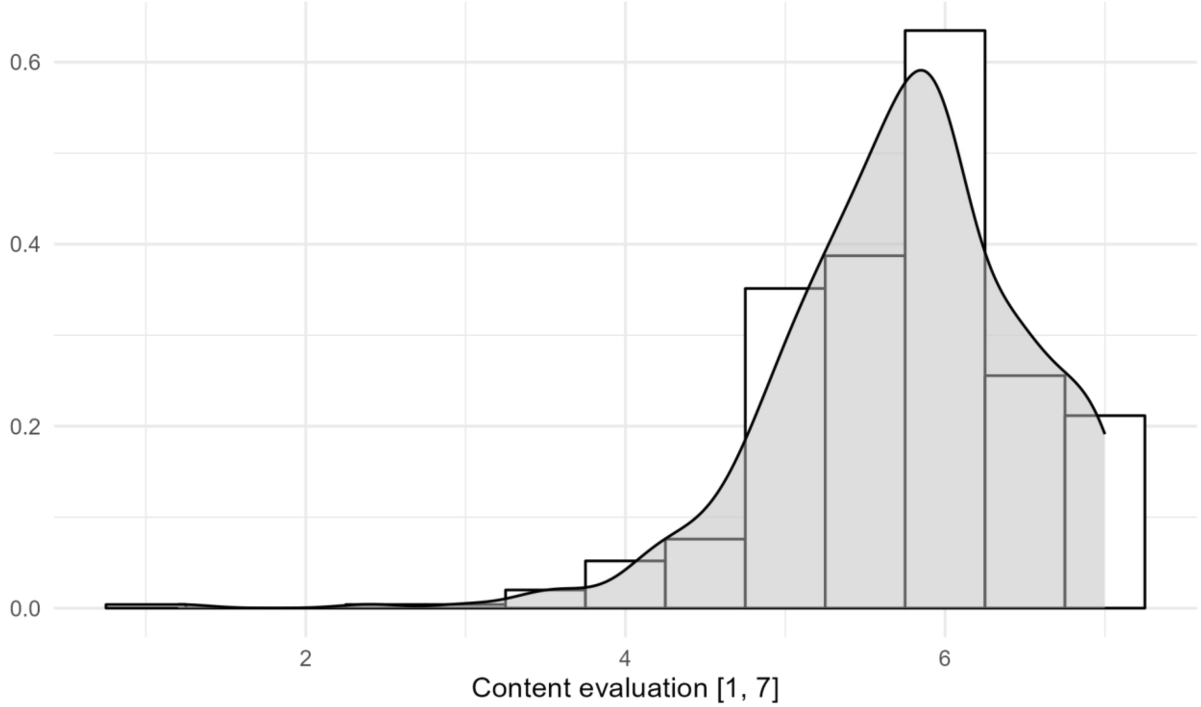

Supplementary Figure 12

Distribution of responses for the outcome variable Intention to share the content in Study 2a (N = 501).

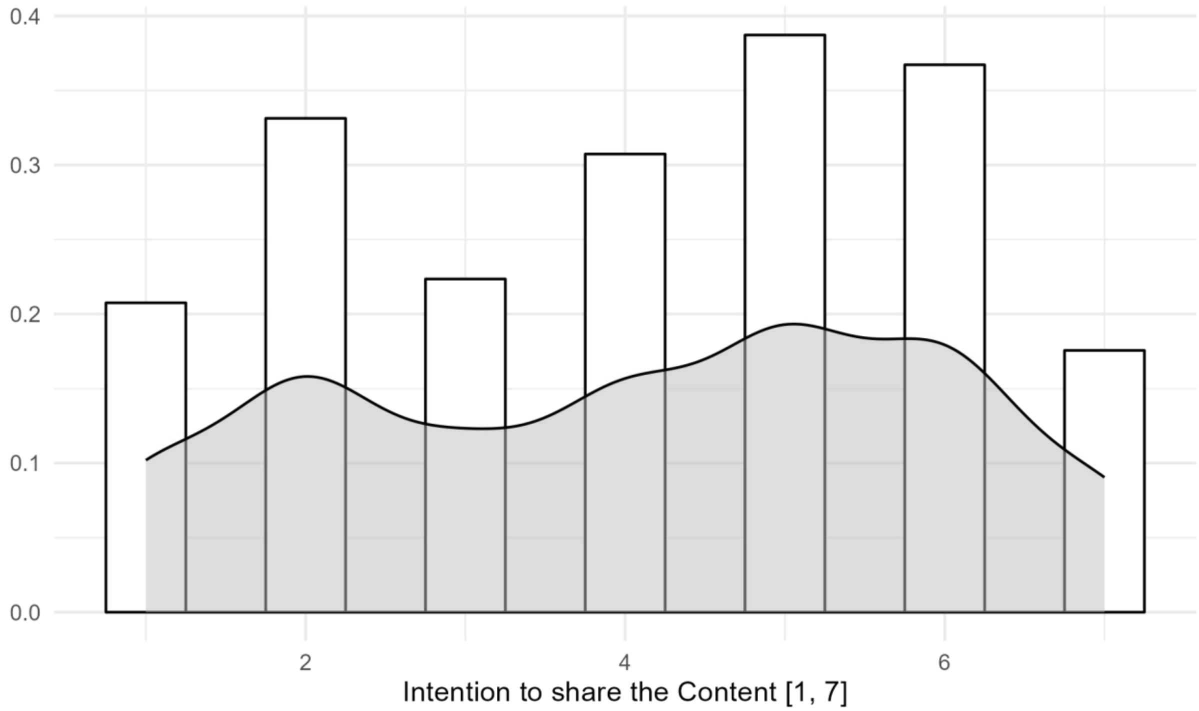

Supplementary Figure 13

Distribution of responses for the outcome variable Intention to follow the advice in Study 2a (N = 501).

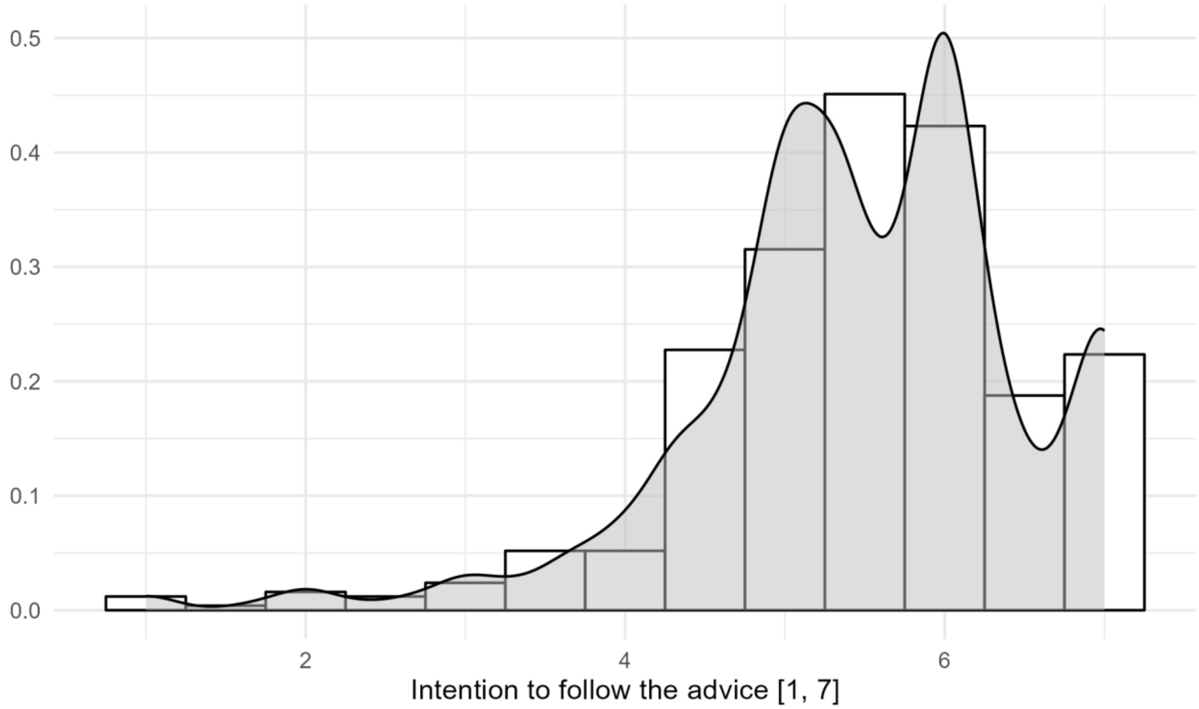

Supplementary Figure 14

Distribution of responses for the outcome variable Author evaluation in Study 2b (*N* = 800).

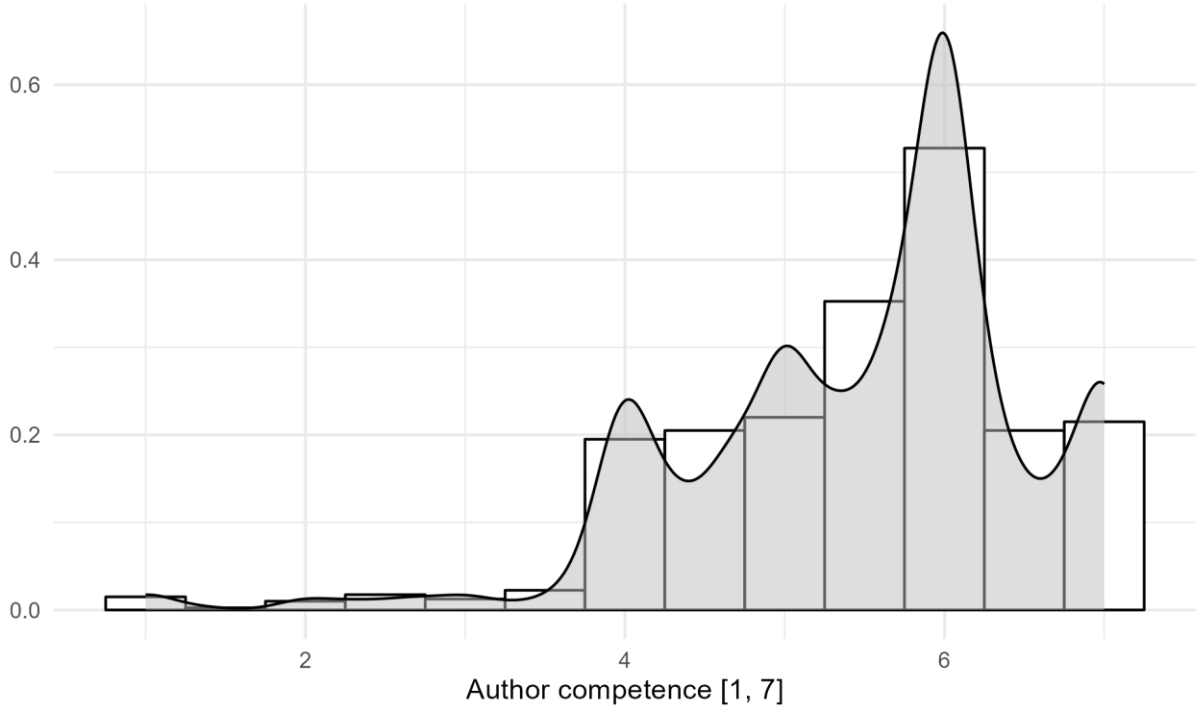

Supplementary Figure 15

Distribution of responses for the outcome variable Content evaluation in Study 2b (N = 800).

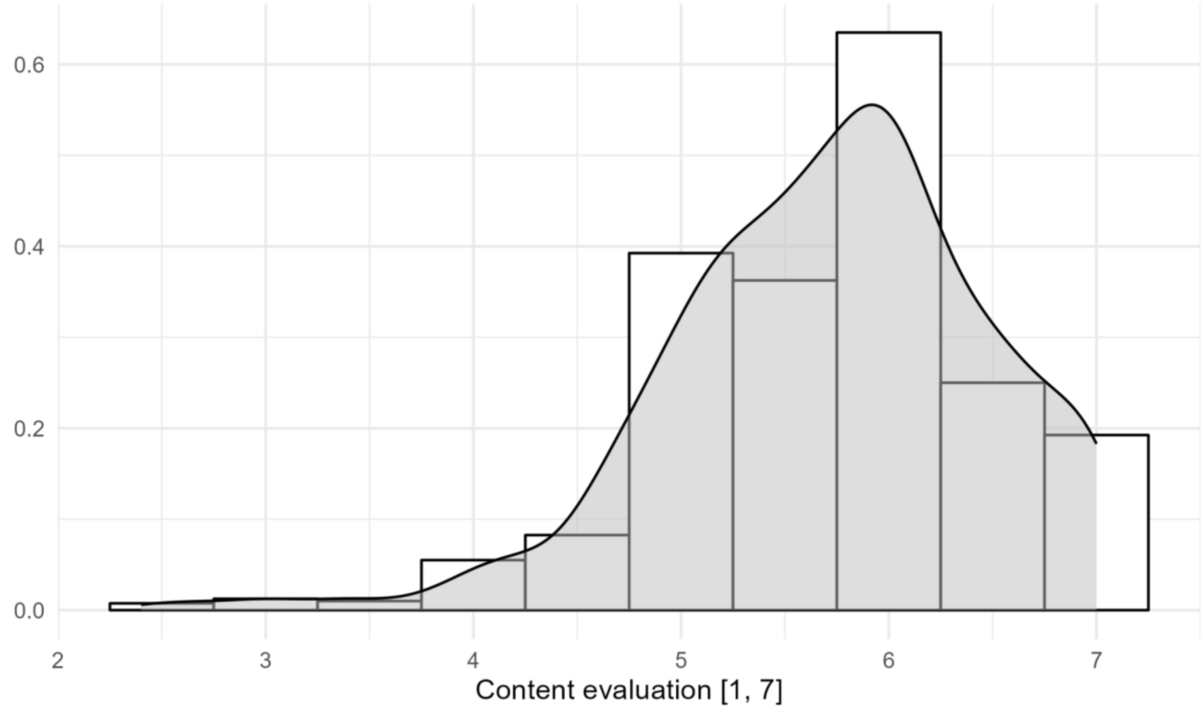

Supplementary Figure 16

Distribution of responses for the outcome variable Intention to share the content in Study 2b (N = 800).

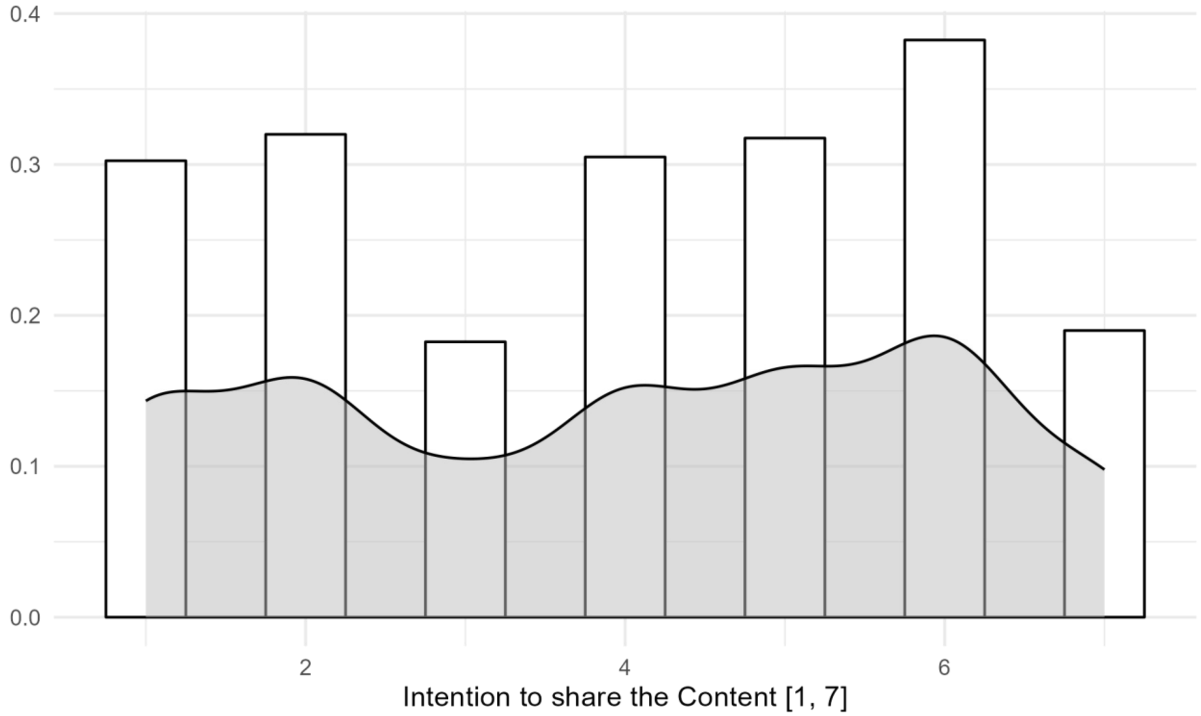

Supplementary Figure 17

Distribution of responses for the outcome variable Intention to follow the advice in Study 2b (*N* = 800).

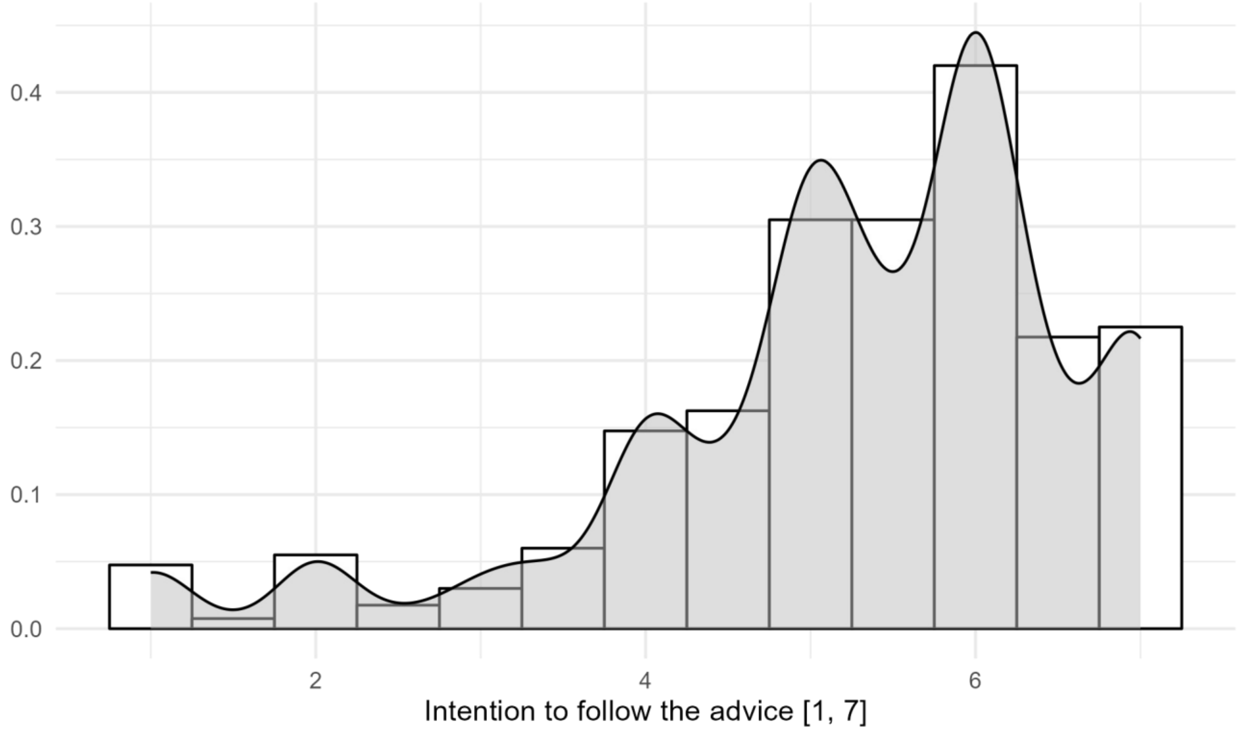

Study 3

Supplementary Figure 18

Distribution of responses for the outcome variable Author evaluation in Study 3 (N = 1004).

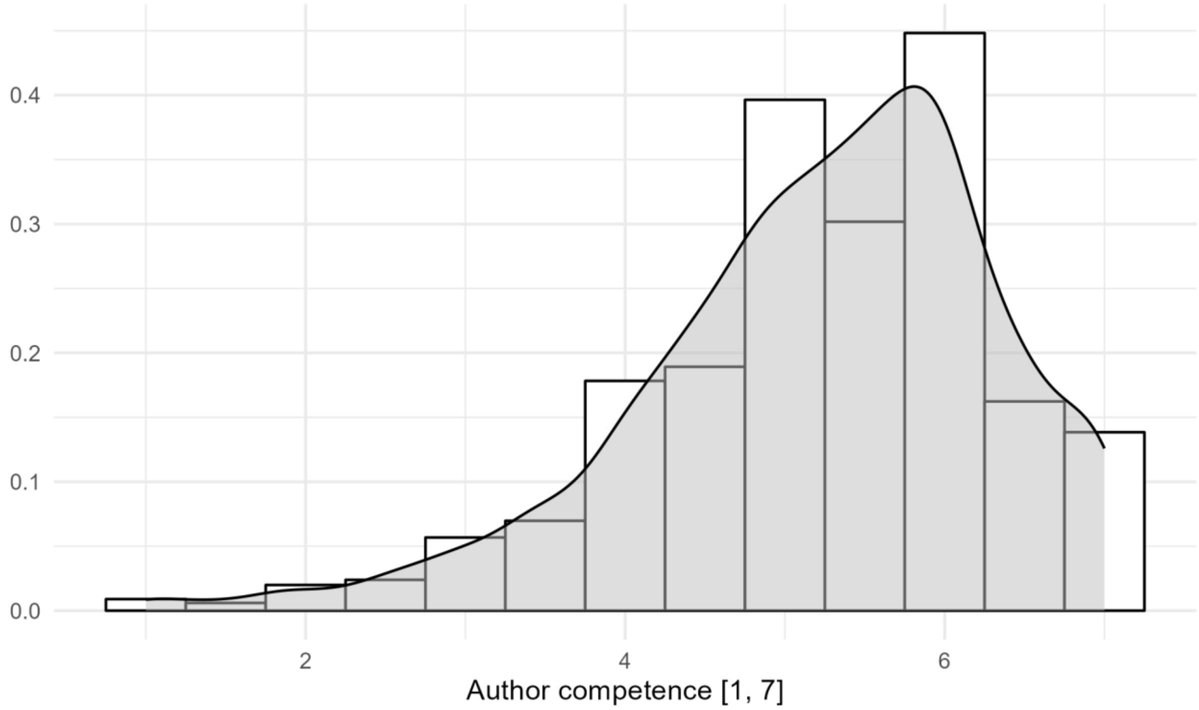

Supplement: Supplementary file 1 — Supplementary Information [file 44271_2023_32_MOESM1_ESM.pdf]
