# Supplementary material for: People devalue generative AI’s competence but not its advice in addressing societal and personal challenges
Source: Commun Psychol. 2023 Nov 15;1:32. doi: 10.1038/s44271-023-00032-x (PMC11332189; doi:10.1038/s44271-023-00032-x)
Supplement: Supplementary file 2 — Reporting Summary [file 44271_2023_32_MOESM2_ESM.pdf]

nature portfolio

Peer Review File

People devalue generative AI’s competence but not its advice in addressing societal and personal challenges

Open Access This file is licensed under a Creative Commons Attribution 4.0 International License, which permits use, sharing, adaptation, distribution and reproduction in any medium or format, as long as you give appropriate credit to the original author(s) and the source, provide a link to the Creative Commons license, and indicate if changes were made. In the cases where the authors are anonymous, such as is the case for the reports of anonymous peer reviewers, author attribution should be to 'Anonymous Referee' followed by a clear attribution to the source work. The images or other third party material in this file are included in the article’s Creative Commons license, unless indicated otherwise in a credit line to the material. If material is not included in the article’s Creative Commons license and your intended use is not permitted by statutory regulation or exceeds the permitted use, you will need to obtain permission directly from the copyright holder. To view a copy of this license, visit http://creativecommons.org/licenses/by/4.0/.

Web links to the author’s journal account have been redacted from the decision letters as indicated to maintain confidentiality

30th Mar 23

Dear Robert,

Thank you again for your patience during the peer-review process.

Your manuscript titled "Content beats competence: People devalue ChatGPT's perceived competence but not its recommendations" has now been seen by 2 reviewers, whose comments are appended below. You will see that the reviewers are enthusiastic about the research question. However, they have raised quite substantial concerns that must be addressed. In light of these comments, we cannot accept the manuscript for publication in its present form, but would be very interested in considering a revised version that fully addresses the referees' concerns and editorial requests.

The reviewers' concerns fall into 3 categories: strength of evidence, treatment of the literature, and presentation of the work (in text and figures).
Please bear in mind that we will be reluctant to approach the reviewers again in the absence of substantial revisions addressing all of these issues, in addition to the editorial requirements below.

Editorially, we consider it necessary that your revision addresses point #7 in Reviewer #1's report through additional empirical data. Following further consultation with the reviewer, we ask you to ideally conduct a new study in which participants are either informed correctly about the origin of a text (human/AI) or temporarily deceived (with later debrief) to understand whether origin and assumed origin have separable effects. Alternative additional studies could consist of a replication of the existing design with an additional assessment of qualitative differences between the text by the participants, or finally, a conceptual replication that is preceded by an independent rating qualitative differences between sample texts, with stimulus material chosen (matched) based on these prior ratings.

Further, as you will see from the information below (including a link to our template and checklist for resubmission), the journal employs strict guidelines for statistics use and reporting. Please make sure you comply with these guidelines, adding Bayesian statistics or two sided tests of equivalence in all instances where you report and subsequently interpret null results.

Among the presentational issues mentioned by the reviewers is the ChatGPT-generated opening paragraph. First, for transparency reasons, we ask that you declare in the Introduction (where you make transparent the origin of the text), which exact prompt was fed to ChatGPT. Second, according to our policy, while large language models may be used as a Method, they cannot be attributed authorship (https://www.nature.com/nature/for-authors/initial-submission): "Large Language Models (LLMs), such as ChatGPT, do not currently satisfy our authorship criteria. Notably an attribution of authorship carries with it accountability for the work, which cannot be effectively applied to LLMs. Use of an LLM should be properly documented in the Methods section of the manuscript". We therefore ask you to remove mention of ChatGPT from the author contribution statement. In your Methods section please mention the use of ChatGPT to generate the opening paragraph in the Introduction, as well as its use to generate stimulus material (with prompts). Third, we invite you to include reflection on the referees' concerns about the quality of the automated text in the Discussion.

We would appreciate it if you could keep us informed about an estimated timescale for resubmission, to facilitate our planning.

We are committed to providing a fair and constructive peer-review process. Please do not hesitate to contact us if you wish to discuss the revision in more detail.

Please use the following link to submit your revised manuscript, point-by-point response to the Reviewers' comments with a list of your changes to the manuscript text (which should be in a separate document to any cover letter) and any completed checklist:

[link redacted]

** This url links to your confidential home page and associated information about manuscripts you may have submitted or be reviewing for us. If you wish to forward this email to co-authors, please delete the link to your homepage first **

Please do not hesitate to contact me if you have any questions or would like to discuss the required revisions further. Thank you for the opportunity to review your work.

Best wishes

Marike

Marike Schiffer, PhD
Chief Editor
Communications Psychology

EDITORIAL POLICIES AND FORMATTING

We ask that you ensure your manuscript complies with our editorial policies. Please ensure that the following formatting requirements are met, and any checklist relevant to your research is completed and uploaded as a Related Manuscript file type with the revised article.

Editorial Policy: Policy requirements (Download the link to your computer as a PDF.)

Furthermore, please align your manuscript with our format requirements, which are summarized on the following checklist:
Communications Psychology formatting checklist

and also in our style and formatting guide Communications Psychology formatting guide .

* TRANSPARENT PEER REVIEW: Communications Psychology uses a transparent peer review system. This means that we publish the editorial decision letters including Reviewers' comments to the authors and the author rebuttal letters online as a supplementary peer review file. However, on author request, confidential information and data can be removed from the published reviewer reports and rebuttal letters prior to publication. If your manuscript has been previously reviewed at another journal, those Reviewers' comments would not form part of the published peer review file.

* CODE AVAILABILITY: All Communications Psychology manuscripts must include a section titled "Code Availability" at the end of the methods section. In the event of publication, we require that the custom analysis code supporting your conclusions is made available in a publicly accessible repository; please choose a repository that provides a DOI for the code; the link to the repository and the DOI must be included in the Code Availability statement. Publication as Supplementary Information will not suffice. We ask you to prepare and upload code at this stage, to avoid delays later on in the process.

* DATA AVAILABILITY:
All Communications Psychology research manuscripts must include a section titled "Data Availability" at the end of the Methods section or main text (if no Methods). More information on this policy, is available at http://www.nature.com/authors/policies/data/data-availability-statements-data-citations.pdf.

At a minimum the Data availability statement must explain how the data can be obtained and whether there are any restrictions on data sharing. Communications Psychology strongly endorses open sharing of da. If you do make your data openly available, please include in the statement:
- Unique identifiers (such as DOIs and hyperlinks for datasets in public repositories)
- Accession codes where appropriate
- If applicable, a statement regarding data available with restrictions
- If a dataset has a Digital Object Identifier (DOI) as its unique identifier, we strongly encourage including this in the Reference list and citing the dataset in the Data Availability Statement.

We recommend submitting the data to discipline-specific, community-recognized repositories, where possible and a list of recommended repositories is provided at http://www.nature.com/sdata/policies/repositories.

If a community resource is unavailable, data can be submitted to generalist repositories such as figshare or Dryad Digital Repository. Please provide a unique identifier for the data (for example a DOI or a permanent URL) in the data availability statement, if possible. If the repository does not provide identifiers, we encourage authors to supply the search terms that will return the data. For data that have been obtained from publicly available sources, please provide a URL and the specific data product name in the data availability statement. Data with a DOI should be further cited in the methods reference section.

Please refer to our data policies at <a

href="http://www.nature.com/authors/policies/availability.html">http://www.nature.com/authors/policies/availability.html.

REVIEWER EXPERTISE:
Reviewer #1: social decision making, human-robot interaction
Reviewer #2: social decision making, human-robot interaction

Reviewer #1 (Remarks to the Author):

Review for "Content beats competence: People devalue ChatGPT's perceived competence but not its recommendations"
The paper "Content beats competence: People devalue ChatGPT's perceived competence but not its recommendations" deals with the increasingly important question of how people evaluate the advice they receive from large language models like ChatGPT.
It is a creative way to begin the paper about ChatGPT by using output from ChatGPT (although it did directly jump at me that the opening paragraph makes sweeping statements without providing the needed references). In general, the paper is well-written and fun to read.
The current version of the manuscript does, however, also have several limitations.

1. The paper would benefit from a stronger embedding in the literature on advice-giving. How do the findings inform that literature?
Also, the emergent literature on AI advice might be useful to discuss, especially for the results of Study 2 on people's stated willingness to follow advice. Some papers to consider are:
Lanz, L., Briker, R., & Gerpott, F. H. (2023). Employees Adhere More to Unethical Instructions from Human Than AI Supervisors: Complementing Experimental Evidence with Machine Learning. Journal of Business Ethics, 1-22.
Leib, M., Köbis, N. C., Rilke, R. M., Hagens, M., & Irlenbusch, B. (2021, Feb 15). The corruptive force of AI-generated advice. Retrieved from http://arxiv.org/pdf/2102.07536v1
2. On page 5 the authors state "advice depends on the identity relevance of the respective context17, with some studies showing even an appreciation of algorithmic advice in contexts with low identity relevance (e.g., certain estimation tasks;18)." Please specify which emotional tasks you are referring to.
3. On page 6, the authors state "We assess several outcome criteria, including evaluations of the author and the content, as well as potential behavioral consequences of these evaluations, that is, sharing the text with others and the intention to follow the recommendation." Speaking of behavioral consequences is not warranted since the authors merely use stated views. Please rephrase.
4. The method of using multiple contexts and, more specifically, the choice of the contexts used are not explained well. Currently, these appear a bit arbitrary. Please briefly explain why these contexts were chosen. The results were hard to follow. For example, the findings for sharing intentions in Study 1 are hard to find & Where are the results for the beliefs about the author of the text? The results could be streamlined to make it easier for the reader to follow. Why not report results for each of the DVs one after the other in separate (short) paragraphs?
5. Figure 1 could be improved too. I find the y-axis description of author evaluation too broad and not very meaningful and suggest relabeling it to "author competence" if I understand it correctly that this is what is plotted (and potentially remind the reader what high values correspond to). If it is

an aggregate measure of all three DVs then this needs to be clarified.
6. In line with the research cited in the introduction, the results differ across contexts. But these findings are not discussed in detail. Please discuss.
7. In both studies, the author of the advice text is manipulated (either human or AI). But due to the fact that actual AI and human advice texts are used, the advice texts might be qualitatively different. This difference in the quality of the texts might potentially drive the observed effects.
8. One criticism of the methods in Study 2 is that by allowing people to select a topic, selection biases emerge. Please explain why you chose this method and whether selection bias is a concern.
9. It appears that there is a deviation between the pre-registration and the actual analysis. Namely, while the authors pre-registered a MANOVA, they ended up conducting multiple ANOVAs. Please highlight all deviations from the pre-registration, preferably in a separate section.

Reviewer #2 (Remarks to the Author):

1. The first paragraph in the introduction is more focused on predictive AI rather than LLMs/generative AI. I understand that it is a nice idea that you got ChatGPT to write it but it didn't do a good job given what the focus of your article is. Your article focuses on generative text, so an intro with "forecasting severe weather events, managing renewable energy resources" is not relevant because LLMs would not suit those types of tasks.

2. My interpretation of the literature that you review in paragraph one of the "related literature" isn't that "People are not particularly good at differentiating between AI- versus human-generated content", but rather that there are still a relatively small number of studies that have explored this which is why your study is important and timely.

3. I don't think that paragraph 2 of "related literature" is an accurate depiction of what "algorithm aversion" is. Algorithm aversion is not related to "content" made by AI (as is the case with generative AI) but as you say later to "advice". Algorithm aversion is related to a different AI paradigm. Similar to how your first paragraph in the intro talks about examples of predictive and decision-making AI models, this paragraph is making a similar mistake. The reviewed literature is more relevant to a different type of AI paradigm. I am not necessarily sure that you can extrapolate the human-AI interaction dynamics present in algorithm aversion for advice based systems, to the generative systems you are exploring here.

4. Altogether I think that most of your Introduction needs rewriting

5. I wouldn't call algorithm aversion "pessimistic". It's a finding, not a normative claim.

6. Agree with the methods, results and discussion for study 1 and 2

7. Your discussion on capability in the general discussion might want to include how this relates to AI resposibility. If we have a lower expectation of AI for a particular context we might hold it as less resposible. The opposite might be true if we have high exprectations. Would recommend this

https://dl.acm.org/doi/abs/10.1145/3514094.3534140 "Causal Framework of Artificial Autonomous Agent Responsibility" as a primer on the topic.

RESPONSE LETTER COMMSPSYCHOL-23-0021

<u>COMMENTS BY THE EDITOR</u>

Editor, Comment 1

Your manuscript titled "Content beats competence: People devalue ChatGPT's perceived competence but not its recommendations" has now been seen by 2 reviewers, whose comments are appended below. You will see that the reviewers are enthusiastic about the research question. However, they have raised quite substantial concerns that must be addressed. In light of these comments, we cannot accept the manuscript for publication in its present form, but would be very interested in considering a revised version that fully addresses the referees' concerns and editorial requests.

The reviewers' concerns fall into 3 categories: strength of evidence, treatment of the literature, and presentation of the work (in text and figures).
Please bear in mind that we will be reluctant to approach the reviewers again in the absence of substantial revisions addressing all of these issues, in addition to the editorial requirements below.

> We are grateful for the reviewers' thoughtful comments that helped us to revise the manuscript. In our revision, we addressed all the comments raised and complied with the editorial requirements. Moreover, we added two additional studies to further strengthen the robustness and implications of our research.

Editor, Comment 2

Editorially, we consider it necessary that your revision addresses point #7 in Reviewer #1's report through additional empirical data. Following further consultation with the reviewer, we ask you to ideally conduct a new study in which participants are either informed correctly about the origin of a text (human/AI) or temporarily deceived (with later debrief) to understand whether origin and assumed origin have separable effects. Alternative additional studies could consist of a replication of the existing design with an additional assessment of qualitative differences between the text by the participants, or finally, a conceptual replication that is preceded by an independent rating qualitative differences between sample texts, with stimulus material chosen (matched) based on these prior ratings.

> This comment (Reviewer 1, Comment 9 according to the structure in this response letter) appears to be based on a misunderstanding of our design in Study 2. In this study, we only rely on AI-generated content but vary whether author identity is known vs. unknown to participants. Hence, actual differences in the quality of human vs. AI advice cannot drive the results. Importantly, in Study 2 we conceptually replicate the results of Study 1, that is, when people know that the content was generated by an AI author, they do not devalue the quality of the content but they devalue the author's competence.

To avoid such a potential misunderstanding and corresponding interpretations of the studies' results among readers, we added a statement/discussion on this issue in the Discussion section (p. 28):

"Secondly, we made efforts to ensure equivalent expert/competence framing across the AI and human conditions in Study 1, as differences in framing may be a potential source of algorithm aversion[26]. However, it is possible that even slight variations in framing may have influenced our results. Relatedly, there may also be objective differences in the quality of the content generated by AI- vs. human-generated content. Although we found no clear pattern of evidence regarding whether the content generated by AI vs. human authors is evaluated differently, this is of course only a snapshot of the specific contexts, prompts, and generative AI models used in these studies. It is important to note, however, that our main findings are based on comparisons between transparent versus non-transparent author identity within the AI author condition (in fact, Studies 2a and 2b did not even include human-generated content), which employed consistent content and framing throughout."

Importantly, we also conducted two new studies—now reported as Studies 2b and 3 in the revised manuscript—to further strengthen the robustness of our findings and their implications. We hope you and the reviewers agree that these additional studies make the manuscript even stronger. Please see our responses to Reviewer 1, Comment 10 (for Study 2b) and to Reviewer 2, Comment 3 (for Study 3), where we summarize the idea and aim of these new studies as well as their results.

Further, as you will see from the information below (including a link to our template and checklist for resubmission), the journal employs strict guidelines for statistics use and reporting. Please make sure you comply with these guidelines, adding Bayesian statistics or two sided tests of equivalence in all instances where you report and subsequently interpret null results.

We now report additional equivalence tests in all instances where we report null results. Here, we consistently test against small effects (based on standard conventions regarding small effect sizes; Cohen, 1988), thus, reporting whether a null effect does or does not allow to reject a small effect.

Among the presentational issues mentioned by the reviewers is the ChatGPT-generated opening paragraph. First, for transparency reasons, we ask that you declare in the Introduction (where you make transparent the origin of the text), which exact prompt was fed to ChatGPT. Second, according to our policy, while large language models may be used as a Method, they cannot be attributed authorship (https://www.nature.com/nature/for-authors/initial-submission): "Large Language Models (LLMs), such as ChatGPT, do not currently satisfy our authorship criteria. Notably an attribution of authorship carries with it accountability for the work, which cannot be effectively applied to LLMs. Use of an LLM should be properly documented in the Methods section of the manuscript". We therefore ask

you to remove mention of ChatGPT from the author contribution statement. In your Methods section please mention the use of ChatGPT to generate the opening paragraph in the Introduction, as well as its use to generate stimulus material (with prompts). Third, we invite you to include reflection on the referees' concerns about the quality of the automated text in the Discussion.

> In response to reviewer 2's concern regarding the quality of the introductory paragraph, we have built a better prompt and now report a revised (and more appropriate) introductory paragraph. We report the exact prompt used to generate the introductory paragraph (p. 3). Furthermore, we removed the mention of ChatGPT from the author contribution statement. Instead, we added sections to the Method sections to declare how and which generative AI was used to create study materials. Because of the large amount of AI-generated content used in the studies (i.e., different contexts), we refer to the online supplement for the specific prompts and outputs.

COMMENTS BY REVIEWER 1

Reviewer 1, Comment 1

The paper "Content beats competence: People devalue ChatGPT's perceived competence but not its recommendations" deals with the increasingly important question of how people evaluate the advice they receive from large language models like ChatGPT.
It is a creative way to begin the paper about ChatGPT by using output from ChatGPT (although it did directly jump at me that the opening paragraph makes sweeping statements without providing the needed references). In general, the paper is well-written and fun to read.
The current version of the manuscript does, however, also have several limitations.

> We thank the reviewer for the positive overall evaluation and the helpful comments to further improve the manuscript. We addressed all of them (for details, see below).

Reviewer 1, Comment 2

The paper would benefit from a stronger embedding in the literature on advice-giving. How do the findings inform that literature?
Also, the emergent literature on AI advice might be useful to discuss, especially for the results of Study 2 on people's stated willingness to follow advice. Some papers to consider are:
Lanz, L., Briker, R., & Gerpott, F. H. (2023). Employees Adhere More to Unethical Instructions from Human Than AI Supervisors: Complementing Experimental Evidence with Machine Learning. Journal of Business Ethics, 1-22.
Leib, M., Köbis, N. C., Rilke, R. M., Hagens, M., & Irlenbusch, B. (2021, Feb 15). The corruptive force of AI-generated advice. Retrieved from http://arxiv.org/pdf/2102.07536v1

> We agree with the reviewer that particularly the literature on advice-giving/taking is relevant to our studies. As suggested, we have extended this section in the Introduction, adding a more thorough review of the previous literature on AI (vs.

human) advice and its psychological effects, including the suggested references as well as additional ones (p. 5).

Reviewer 1, Comment 3

On page 5 the authors state "advice depends on the identity relevance of the respective context17, with some studies showing even an appreciation of algorithmic advice in contexts with low identity relevance (e.g., certain estimation tasks;18)." Please specify which emotional tasks you are referring to.

We now provide specific examples of the advice contexts throughout this section. In this particular example, we specify: "[…] with some studies even showing an appreciation of algorithmic advice in contexts with low identity relevance, such as numerical estimation or forecasting tasks[20]."

Reviewer 1, Comment 4

On page 6, the authors state "We assess several outcome criteria, including evaluations of the author and the content, as well as potential behavioral consequences of these evaluations, that is, sharing the text with others and the intention to follow the recommendation." Speaking of behavioral consequences is not warranted since the authors merely use stated views. Please rephrase.

We thank the reviewer for this important remark. We made sure to always refer to "behavioral intentions" throughout the manuscript. The specific sentence referred to by the reviewer was rewritten as follows (p. 6):

"We assess several outcome criteria, including evaluations of the author and the content, as well as its potential downstream consequences, such as the choice between receiving AI- vs. human-generated advice."

Reviewer 1, Comment 5

The method of using multiple contexts and, more specifically, the choice of the contexts used are not explained well. Currently, these appear a bit arbitrary. Please briefly explain why these contexts were chosen.

We added our reasoning for why the specific contexts were chosen in the respective Method sections. The relevant section for Study 1 reads as follows (p. 8):

"These specific contexts were selected based on their identification as pressing challenges by global institutions such as the European Union or the United Nations, as well as the availability of corresponding expert recommendations that were also suited as prompts for generative AI (see above)."

The relevant section for Studies 2a and 2b now reads as follows (p. 12):

> "The contexts included in this study—exercising more regularly, quitting smoking, eating healthier, reducing time spent on the phone, saving money, having a positive impact on the world—were chosen based on the authors' internal discussion, aiming to include personal challenges that would be relevant for many individuals from the target population."
>
> Despite covering a wide range of contexts, we agree that this selection may appear somewhat arbitrary. Therefore, we added this as a limitation to the Discussion section (p. 28):
>
> "It is important to acknowledge the limitations of our research. Firstly, while we examined a variety of contexts related to societal and personal challenges, we naturally cannot claim generalizability of our findings to other contexts."

Reviewer 1, Comment 6

The results were hard to follow. For example, the findings for sharing intentions in Study 1 are hard to find & Where are the results for the beliefs about the author of the text? The results could be streamlined to make it easier for the reader to follow. Why not report results for each of the DVs one after the other in separate (short) paragraphs?

> We thank the reviewer for this constructive suggestion. We realized that the initial reporting of the results was hard to follow. To improve readability, we have restructured the presentation of the results and now report the results for each outcome measure subsequently. Naturally, the reporting of the significant effects is more comprehensive, incorporating subsequent robustness checks. Additionally, we now provide a summary sentence at the end of the confirmatory results section, clarifying which outcomes supported the hypothesis and which one not (p. 11):
>
> "Taken together, these results indicate that although people exhibit AI aversion in terms of perceived author competence, this aversion does not extend to their evaluation of the content quality or their sharing intentions. This finding suggests that people generally like recommendations to address pressing societal challenges generated by AI as much as those generated by human experts, although they clearly devaluate the competence of AI advisors."

Reviewer 1, Comment 7

Figure 1 could be improved too. I find the y-axis description of author evaluation too broad and not very meaningful and suggest relabeling it to "author competence" if I understand it correctly that this is what is plotted (and potentially remind the reader what high values correspond to). If it is an aggregate measure of all three DVs then this needs to be clarified.

As suggested, we have changed the label of the y-axis in Figures 1 and 2 to "author competence." Furthermore, we added to the figure captions how the outcome variable was created.

Reviewer 1, Comment 8

In line with the research cited in the introduction, the results differ across contexts. But these findings are not discussed in detail. Please discuss.

There are indeed some descriptive differences in the responses to different contexts, particularly in Study 2a. However, the test power is too low to make definite conclusions about the nature of these differences, i.e., whether this is due to random variation or systematic differences due to the characteristics of the contexts.

That being said, in the newly conducted Study 2b—which was identical to Study 2a except for the exogenous assignment of context to participants (see our response to your Comment 10, below)—we did not replicate these (minor) differences. That is, the hypothesized pattern on our main outcome measure (author competence) was descriptively present across all contexts. Therefore, we believe it is appropriate to refrain from discussing such non-robust differences.

Reviewer 1, Comment 9

In both studies, the author of the advice text is manipulated (either human or AI). But due to the fact that actual AI and human advice texts are used, the advice texts might be qualitatively different. This difference in the quality of the texts might potentially drive the observed effects.

In Study 1, the texts presented to participants were indeed either generated by a human expert or by generative AI. However, there is no indication that participants evaluated the quality of the texts differently (as reported in the Results section of Study 1, p. 11). Moreover, when participants were not aware of the author's identity (non-transparent condition), the human- vs. AI-generated text was not more likely to be recognized as human- vs. AI-generated text (see p. 10/11).

It is important to note that in Study 2, we only used AI-generated texts for participants to evaluate. Hence, any difference in the evaluation of the author competence stems solely from whether participants knew (vs. did not know) the author identity of the AI author. In other words, this study not only conceptually replicates the findings from Study 1, but it also rules out the possibility that the difference can be attributed to varying qualities of human- vs. AI-generated text.

As this important feature of Study 2 was obviously not clearly articulated, we now added a sentence to the Discussion section emphasizing this, but also acknowledging that such effects may depend on the specific study materials (p. 28):

> "Relatedly, there may also be objective differences in the quality of the content generated by AI- vs. human-generated content. Although we found no clear pattern of evidence regarding whether the content generated by AI vs. human authors is evaluated differently, this is of course only a snapshot of the specific contexts, prompts, and generative AI models used in these studies. It is important to note, however, that our main findings are based on comparisons between transparent versus non-transparent author identity within the AI author condition (in fact, Studies 2a and 2b did not even include human-generated content), which employed consistent content and framing throughout."

Reviewer 1, Comment 10
One criticism of the methods in Study 2 is that by allowing people to select a topic, selection biases emerge. Please explain why you chose this method and whether selection bias is a concern.

> We agree that selection bias could have occurred in Study 2. However, it was the deliberate aim of Study 2 to let participants choose a personal challenge they identify with. Otherwise, one may argue, they might be less sensitive to perceiving differences in the materials.
>
> Nevertheless, as we cannot exclude the possibility that selection bias has unintended effects that might have affected the results, we decided to replicate the exact same study, with the only difference being the random assignment of context to participants as an exogenous manipulation. This additional study is now reported as Study 2b in the manuscript (whereas the original study is reported as Study 2a). Study 2b is reported on p. 18-20 in the revised manuscript. Importantly, this new study qualitatively replicates the results even with the use of random context assignment, providing further support to our conclusions.

Reviewer 1, Comment 11
It appears that there is a deviation between the pre-registration and the actual analysis. Namely, while the authors pre-registered a MANOVA, they ended up conducting multiple ANOVAs. Please highlight all deviations from the pre-registration, preferably in a separate section.

> We thank the reviewer for noting this. We have added the pre-registered MANOVA to the Results section of Study 1. Note that we did not preregister to conduct a MANOVA for the other studies.

COMMENTS BY REVIEWER 2

Reviewer 2, Comment 1
The first paragraph in the introduction is more focused on predictive AI rather than LLMs/generative AI. I understand that it is a nice idea that you got ChatGPT to write it but it didn't do a good job given what the focus of your article is. Your article focuses on generative

text, so an intro with "forecasting severe weather events, managing renewable energy resources" is not relevant because LLMs would not suit those types of tasks.

We agree that some aspects of the introductory paragraph provided examples that are not within the scope of the present research. Therefore, we wrote a new prompt to generate a more appropriate introductory paragraph using ChatGPT. The examples given in this new paragraph only deal with intelligent suggestions, such as the one investigated in this research (p. 3; based on an editorial request we present the prompt in the text underneath the introductory paragraph):

“The growing integration of artificial intelligence (AI) into various aspects of our lives presents a remarkable opportunity to address both societal and personal challenges. AI’s potential lies not only in its ability to analyze data but also in its capacity to provide intelligent suggestions to tackle complex problems. In the realm of societal challenges, such as climate change and pandemic preparedness, AI can contribute by offering proactive recommendations for sustainable practices and efficient response strategies. Additionally, in the context of personal challenges like improving healthy eating and saving money, AI can act as a personalized advisor, providing tailored suggestions and guidance. By leveraging AI’s capability to generate intelligent suggestions, we can empower individuals and societies to navigate these challenges more effectively, fostering sustainable behavior and improving overall well-being.”

Reviewer 2, Comment 2

My interpretation of the literature that you review in paragraph one of the "related literature" isn't that "People are not particularly good at differentiating between AI- versus human-generated content", but rather that there are still a relatively small number of studies that have explored this which is why your study is important and timely.

We agree that prior research on the topic is rather scarce and, accordingly, adapted the first sentence of the Related Literature section (p. 4):

“Although the available evidence is rather limited, prior research indicates that people are not particularly good at differentiating between AI- versus human-generated content (e.g., stories, news articles, recipes, poems) when the author identity is not transparent—even before recent improvements in generative AI as build into ChatGPT[4,5].”

Reviewer 2, Comment 3

I don't think that paragraph 2 of "related literature" is an accurate depiction of what "algorithm aversion" is. Algorithm aversion is not related to "content" made by AI (as is the case with generative AI) but as you say later to "advice". Algorithm aversion is related to a different AI paradigm. Similar to how your first paragraph in the intro talks about examples of predictive and decision-making AI models, this paragraph is making a similar mistake.

The reviewed literature is more relevant to a different type of AI paradigm. I am not necessarily sure that you can extrapolate the human-AI interaction dynamics present in algorithm aversion for advice based systems, to the generative systems you are exploring here.

We thank the reviewer for this thoughtful comment. We agree that previous studies showing algorithm aversion differ in two important aspects from the current research: First, previous studies did not focus on generative AI. Thus, the crucial difference between the previous research and the current research lies in the underlying technology used to generate suggestions for human actors. However, support systems are similar in terms of the nature of the decision support. In both cases, humans receive advice from algorithmic/AI agents. This relates to a second difference between previous and the current research: Studies 1, 2a, and 2b assess participants' evaluations and behavioral intentions related to the (AI- vs. human-generated) advice they receive, whereas previous studies on algorithm aversion mainly focused on the choice between receiving advice by an algorithmic vs. human advisor. This difference makes comparisons to the previous literature on algorithm aversion rather difficult, but it also allows a more fine-grained analysis of the very nature of AI/algorithm aversion.

To better explain the differences to the previous literature, our review of the algorithm aversion literature now starts as follows (p. 5):

"But how do people evaluate content when they know that it has been generated by an AI? Much of the research in this domain has focused on studying individual preferences for AI/algorithm- versus human-generated advice for different tasks."

To further address the reviewer's concern, we aimed to bridge our research to the existing research on AI/algorithm aversion. To this end, we have conducted another preregistered study, now reported as Study 3 in the revised manuscript. In this study, we investigate whether participants would prefer to receive advice on solving personal challenges from a human expert or a knowledgeable AI. Prior to making their decision, all participants received advice on how to solve a different personal challenge from both an AI and a human (in random order). Half of the participants were informed about the identity of the authors, whereas the other half was not. This allowed us to manipulate participants' experience with AI- vs. human-generated content (as people in reality may gain more experience with AI-generated content over time), while ensuring that they received the exact same information for evaluation.

We found clear evidence for a preference for human advisors over AI advisors, replicating an AI/algorithm aversion effect in the context of generative AI. Importantly, those participants who previously learned about the identity of authors and, thus, gained experience with the quality of AI- and human-generated advice,

were more likely to select an AI advisor afterward. This effect was stronger for participants who rated the AI-generated content they had received before more positively in relation to the human-generated content, but only when they also learned about the author identity. Hence, this novel study provides a better connection and comparison to the previous literature on algorithm aversion by showing discriminatory preferences for AI- vs. human-generated advice. Thus, we believe that our studies can be interpreted as an application and extension of the algorithm aversion effect in the context of generative AI when faced with advice on how to address societal or personal challenges. Study 3 is reported on p. 20-25 in the revised manuscript.

Because of the insights gained from Study 3, we revised our contribution statement, emphasizing that the present research includes both evaluations and choice between AI- vs. human-generated advice (p. 5/6):

"Our main contribution is to present one of the first systematic investigations into people's evaluations of advice generated by generative AI and their willingness to receive such advice. We focus on advice in contexts that have some identity relevance to the evaluators. That is, we investigate recommendations to societal challenges (Studies 1 and 3) and personal challenges (Studies 2a and 2b) when evaluators are (vs. are not) aware of the author identity. We assess several outcome criteria, including evaluations of the author and the content, as well as its potential downstream consequences, such as the choice between receiving AI- vs. human-generated advice."
"

Reviewer 2, Comment 4
Altogether I think that most of your Introduction needs rewriting.

Following the suggestions by both reviewers, we carefully rewrote the Introduction section to better link our research to the previous work on algorithm aversion. We are grateful for the suggestions we received.

Reviewer 2, Comment 5
I wouldn't call algorithm aversion "pessimistic". It's a finding, not a normative claim.

We removed this statement from the manuscript.

Reviewer 2, Comment 6
Agree with the methods, results and discussion for study 1 and 2.

We thank the reviewer for the positive evaluation of our reported methods and results. Note that we have expanded these sections by adding two novel studies to strengthen the robustness and conclusions from our findings (Study 2b, see our response to

Reviewer 1, Comment 10; Study 3, see our response to your Comment 3). Furthermore, following an editorial request, we now also report equivalence tests in all instances where we report (and interpret) null results.

Reviewer 2, Comment 7
Your discussion on capability in the general discussion might want to include how this relates to AI responsibility. If we have a lower expectation of AI for a particular context we might hold it as less responsible. The opposite might be true if we have high expectations. Would recommend this https://dl.acm.org/doi/abs/10.1145/3514094.3534140 "Causal Framework of Artificial Autonomous Agent Responsibility" as a primer on the topic.

This is a very interesting thought and relates to our motivation to add Study 3 as a novel study to the manuscript (see above). As the relation between AI's perceived competence and blame for potential negative outcomes due to the AI advice has not been part of our investigation, we added a discussion and call for future research on this relation to the Discussion section (p. 27):

"Although AI-generated advice was not found to be devalued per se, the fact that AI authors were perceived as less competent than human authors when the author identity was known has important implications that can be tested in future research. For instance, some work suggests that higher levels of perceived capability can lead to higher expectations regarding outcomes[24]. Higher expectations, in turn, could lead to more blaming when the outcome does not meet the expectations. Thus, it might be fruitful to investigate the relation between the perceived competence of generative AI, evaluation of the content generated by AI, and blame (oneself vs. AI advisor) for negative outcomes following the content evaluation by others."

17th Aug 23

Dear Robert,

Your manuscript titled "People devalue generative AI’s competence but not its advice in addressing societal and personal challenges" has now been seen by our reviewers, whose comments appear below. In light of their advice I am delighted to say that we are happy, in principle, to publish a suitably revised version in Communications Psychology under the open access CC BY license (Creative Commons Attribution v4.0 International License).

We therefore invite you to revise your paper one last time to address the remaining concerns of our reviewers and a list of editorial requests. At the same time we ask that you edit your manuscript to comply with our format requirements and to maximise the accessibility and therefore the impact of your work.

EDITORIAL REQUESTS:

Please review our specific editorial comments and requests regarding your manuscript in the attached "Editorial Requests Table". Please outline your response to each request in the right hand column. Please upload the completed table with your manuscript files as a Related Manuscript file.

If you have any questions or concerns about any of our requests, please do not hesitate to contact me.

SUBMISSION INFORMATION:

In order to accept your paper, we require the files listed at the end of the Editorial Requests Table; the list of required files is also available at https://www.nature.com/documents/commsj-file-checklist.pdf .

OPEN ACCESS:

Communications Psychology is a fully open access journal. Articles are made freely accessible on publication under a CC BY license (Creative Commons Attribution 4.0 International License). This license allows maximum dissemination and re-use of open access materials and is preferred by many research funding bodies.

For further information about article processing charges, open access funding, and advice and support from Nature Research, please visit https://www.nature.com/commspsychol/article-processing-charges

At acceptance, you will be provided with instructions for completing this CC BY license on behalf of all authors. This grants us the necessary permissions to publish your paper. Additionally, you will be asked to declare that all required third party permissions have been obtained, and to provide billing

information in order to pay the article-processing charge (APC).

* TRANSPARENT PEER REVIEW: Communications Psychology uses a transparent peer review system. On author request, confidential information and data can be removed from the published reviewer reports and rebuttal letters prior to publication. If you are concerned about the release of confidential data, please let us know specifically what information you would like to have removed. Please note that we cannot incorporate redactions for any other reasons.

* CODE AVAILABILITY: All Communications Psychology manuscripts must include a section titled "Code Availability" at the end of the methods section. We require that the custom analysis code supporting your conclusions is made available in a publicly accessible repository at this stage; please choose a repository that generates a digital object identifier (DOI) for the code; the link to the repository and the DOI must be included in the Code Availability statement. Publication as Supplementary Information will not suffice.

* DATA AVAILABILITY:
All Communications Psychology manuscripts must include a section titled "Data Availability" at the end of the Methods section. More information on this policy, is available in the Editorial Requests Table and at http://www.nature.com/authors/policies/data/data-availability-statements-data-citations.pdf.

Please use the following link to submit the above items:
[link redacted]
** This url links to your confidential home page and associated information about manuscripts you may have submitted or be reviewing for us. If you wish to forward this email to co-authors, please delete the link to your homepage first **

We hope to hear from you within two weeks; please let us know if you need more time.

Best regards,

Marike

Marike Schiffer, PhD
Chief Editor
Communications Psychology

REVIEWERS' COMMENTS:

Reviewer #1 (Remarks to the Author):

My comments have been addressed in a satisfactory manner.

A few minor points remain:
1. I suggest careful proof-reading as I spotted a few grammar mistakes / typos,
e.g line 68: "(...) recent improvements in generative AI such as GPT 3.5 and GPT 4 as build into ChatGPT4,5." should probably say "(...) recent improvements in generative AI such as GPT 3.5 and GPT 4 are built into ChatGPT4,5."

line 518: "Nevertheless, AI (vs. humans) are choses less likely" should be "Nevertheless, AI (vs. humans) are chosen less likely"

2. Interpreation of REsults for Study 2a vs. b
The authors focus on the commonalities between both studies, which is generally fine. But I still think they should mention the differences across contexts between Study 2a and b that become apparent when inspecting Figure 2.

Reviewer #2 (Remarks to the Author):

I believe that the Author's have successfully responded to the reviews and that their article is ready for publication

RESPONSE LETTER COMMSPSYCHOL-23-0021A

COMMENTS BY THE EDITOR

Editor, Comment 1

Please review our specific editorial comments and requests regarding your manuscript in the attached "Editorial Requests Table". Please outline your response to each request in the right hand column. Please upload the completed table with your manuscript files as a Related Manuscript file.

> We have followed all the editorial requests and responded to them in the attached "Editorial Requests Table."

COMMENTS BY REVIEWER 1

Reviewer 1, Comment 1

My comments have been addressed in a satisfactory manner.

> We are happy that our revisions are evaluated so positively. We thank the reviewer for the constructive review process.

Reviewer 1, Comment 2

A few minor points remain:

1. I suggest careful proof-reading as I spotted a few grammar mistakes / typos,

e.g line 68: "(...) recent improvements in generative AI such as GPT 3.5 and GPT 4 as build into ChatGPT4,5." should probably say "(...) recent improvements in generative AI such as GPT 3.5 and GPT 4 are built into ChatGPT4,5."

line 518: "Nevertheless, AI (vs. humans) are choses less likely" should be "Nevertheless, AI (vs. humans) are chosen less likely"

> We have corrected these errors/typos and carefully checked the manuscript for further errors.

Reviewer 1, Comment 2

2. Interpreation of REsults for Study 2a vs. b

The authors focus on the commonalities between both studies, which is generally fine. But I still think they should mention the differences across contexts between Study 2a and b that become apparent when inspecting Figure 2.

> We have been careful in interpreting potential differences between contexts (e.g., by adding equivalence tests to all relevant null findings). For the given results regarding the evaluations across contexts in Studies 2a and 2b, we do not think that the results are robust enough to allow conclusions regarding context differences. In fact, in Study 2b we do not find any indication of context differences. Nevertheless, we cannot identify the reason for these differences as shown in Study 2a (note that the effect is not significant though). Therefore, we would prefer to stick to the current interpretation of such (small) differences found only in Study 2a but not in Study 2b

as provided on p. 17:

"However, given that we cannot reject a small effect based on the equivalence test, we refrain from speculations whether the differences between context are due to random variation or are subject to systematic differences related to the nature of the contexts."

COMMENTS BY REVIEWER 2

Reviewer 2, Comment 1

I believe that the Author's have successfully responded to the reviews and that their article is ready for publication

We are happy that our revisions are evaluated so positively. We thank the reviewer for the constructive review process.
