# Supplementary material for: People devalue generative AI’s competence but not its advice in addressing societal and personal challenges
Source: Commun Psychol. 2023 Nov 15;1:32. doi: 10.1038/s44271-023-00032-x (PMC11332189; doi:10.1038/s44271-023-00032-x)
Supplement: Supplementary file 3 — Peer Review File [file 44271_2023_32_MOESM3_ESM.pdf]

nature portfolio

Corresponding author(s): Robert Böhm

Last updated by author(s): Oct 13, 2023

Reporting Summary

Nature Portfolio wishes to improve the reproducibility of the work that we publish. This form provides structure for consistency and transparency in reporting. For further information on Nature Portfolio policies, see our Editorial Policies and the Editorial Policy Checklist.

Statistics

For all statistical analyses, confirm that the following items are present in the figure legend, table legend, main text, or Methods section.

n/a	Confirmed	
☐	☒	The exact sample size (*n*) for each experimental group/condition, given as a discrete number and unit of measurement
☐	☒	A statement on whether measurements were taken from distinct samples or whether the same sample was measured repeatedly
☐	☒	The statistical test(s) used AND whether they are one- or two-sided<br>*Only common tests should be described solely by name; describe more complex techniques in the Methods section.*
☐	☒	A description of all covariates tested
☐	☒	A description of any assumptions or corrections, such as tests of normality and adjustment for multiple comparisons
☐	☒	A full description of the statistical parameters including central tendency (e.g. means) or other basic estimates (e.g. regression coefficient) AND variation (e.g. standard deviation) or associated estimates of uncertainty (e.g. confidence intervals)
☐	☒	For null hypothesis testing, the test statistic (e.g. *F*, *t*, *r*) with confidence intervals, effect sizes, degrees of freedom and *P* value noted<br>*Give P values as exact values whenever suitable.*
☒	☐	For Bayesian analysis, information on the choice of priors and Markov chain Monte Carlo settings
☐	☒	For hierarchical and complex designs, identification of the appropriate level for tests and full reporting of outcomes
☐	☒	Estimates of effect sizes (e.g. Cohen's *d*, Pearson's *r*), indicating how they were calculated

Our web collection on statistics for biologists contains articles on many of the points above.

Software and code

Policy information about availability of computer code

Data collection	Participants were recruited via Prolific. The surveys were programmed with Qualtrics.
Data analysis	For statistical analyses, R 4.2.3 was used, including the packages tidyverse (1.3.2), psych (2.2.9), knitr (1.4.1), car (3.1.1), and broom (1.0.2).

For manuscripts utilizing custom algorithms or software that are central to the research but not yet described in published literature, software must be made available to editors and reviewers. We strongly encourage code deposition in a community repository (e.g. GitHub). See the Nature Portfolio guidelines for submitting code & software for further information.

Data

Policy information about availability of data

All manuscripts must include a data availability statement. This statement should provide the following information, where applicable:

- Accession codes, unique identifiers, or web links for publicly available datasets
- A description of any restrictions on data availability
- For clinical datasets or third party data, please ensure that the statement adheres to our policy

The study materials, data, and analysis codes are available on the Open Science Framework at https://osf.io/6ptx8/.

Human research participants

Policy information about studies involving human research participants and Sex and Gender in Research.

Reporting on sex and gender	Participants gender was assessed and is reported in the manuscript. All samples were recruited gender-balanced. Gender is reported as a control variable to all major analyses in the online supplement.
Population characteristics	We recruited participants from the US. The mean age of participants was 41 (SD = 14) in Study 1, 40 (SD = 15) in Study 2a, 40 (SD = 14) in Study 2b, and 42 (SD = 15) in Study 3.
Recruitment	We recruited a gender-balanced convenience sample online via Prolific.
Ethics oversight	The studies received approval from the ethical review board of the Department of Occupational, Economic, and Social Psychology at the University of Vienna, Austria.

Note that full information on the approval of the study protocol must also be provided in the manuscript.

Field-specific reporting

Please select the one below that is the best fit for your research. If you are not sure, read the appropriate sections before making your selection.

☐ Life sciences ☒ Behavioural & social sciences ☐ Ecological, evolutionary & environmental sciences

For a reference copy of the document with all sections, see nature.com/documents/nr-reporting-summary-flat.pdf

Behavioural & social sciences study design

All studies must disclose on these points even when the disclosure is negative.

Study description	All studies are quantitative, experimental survey studies.
Research sample	The studies used online convenience samples of US participants from Prolific. There were no screening criteria but the samples were composed to be gender-balanced. Invitations to each study screened out participants who already participated in the previous studies.
Sampling strategy	Convenience sample; sample sizes were determined via a-priori power analyses as reported in manuscript and in the preregistration protocols (Study 1: https://aspredicted.org/xj2yn.pdf; Study 2a: https://aspredicted.org/h9y6v.pdf; Study 2b: https://aspredicted.org/w86qh.pdf; Study 3: https://aspredicted.org/kp2sb.pdf). Recruitment was stopped by the survey provider as soon as the final sample was collected.
Data collection	All studies were conducted online, hence, there was no experimenter present while participants completed the study.
Timing	Study 1 was collected on January 4, 2023. Study 2a was collected on January 15, 2023. Study 2b was conducted on April 7, 2023. Study 3 was conducted on June 30, 2023.
Data exclusions	No data was excluded from the analyses.
Non-participation	We have no information about dropout during the study.
Randomization	Allocation to the experimental conditions was random except for the quasi-experimental factor in Study 2a as described in manuscript.

Reporting for specific materials, systems and methods

We require information from authors about some types of materials, experimental systems and methods used in many studies. Here, indicate whether each material, system or method listed is relevant to your study. If you are not sure if a list item applies to your research, read the appropriate section before selecting a response.

Materials & experimental systems

n/a	Involved in the study	
☒	☐	Antibodies
☒	☐	Eukaryotic cell lines
☒	☐	Palaeontology and archaeology
☒	☐	Animals and other organisms
☒	☐	Clinical data
☒	☐	Dual use research of concern

Methods

n/a	Involved in the study	
☒	☐	ChIP-seq
☒	☐	Flow cytometry
☒	☐	MRI-based neuroimaging
